# Measurable Residual Disease Testing in Multiple Myeloma Routine Clinical Practice: A Modified Delphi Study

**DOI:** 10.1097/HS9.0000000000000942

**Published:** 2023-08-30

**Authors:** Karthik Ramasamy, Hervé Avet-Loiseau, Cecilie Hveding Blimark, Michel Delforge, Francesca Gay, Salomon Manier, Joaquín Martinez-Lopez, Maria Victoria Mateos, Mohamad Mohty, Niels W.C.J. van de Donk, Katja Weisel

**Affiliations:** 1 Oxford University Hospitals NHS Foundation Trust, Radcliffe Department of Medicine, Oxford University, Oxford, United Kingdom; 2 University Institute of Cancer Toulouse, University Hospital of Toulouse, Toulouse, France; 3 Sahlgrenska University Hospital, Gothenburg, Sweden; 4 University Hospital Leuven, Leuven, Belgium; 5 University of Torino, Turin, Italy; 6 University of Lille, Lille, France; 7 12 de Octubre University Hospital, Madrid, Spain; 8 University Hospital of Salamanca, Salamanca Biomedical Research Institute (IBSAL), CIC, Ciberonc, Salamanca, Spain; 9 Hospital Saint-Antoine, Sorbonne University, INSERM UMRs 938, Paris, France; 10 VU University Medical Center, Amsterdam, Netherlands; 11 University Medical Center Hamburg-Eppendorf, Hamburg, Germany

## Abstract

We used a modified Delphi approach to establish areas of consensus and nonconsensus regarding the utility of determining measurable residual disease (MRD) to assess multiple myeloma (MM) treatment response, which may inform disease management and design of future clinical trials. This modified Delphi study incorporated 2 iterative rounds of surveys to evaluate the opinions of an expert panel of 61 practicing hematological oncologists from across 14 countries in Europe concerning the use of MRD testing in MM management. Survey 1 assessed experts’ opinions on MRD testing in different clinical situations and associated challenges. Survey 2 focused on the lack of consensus areas identified in survey 1. Consensus to an individual question was defined *a priori* as 75% agreement or disagreement by the panel. From the 2 rounds of surveys, the experts reached consensus agreement that MRD testing should be performed in newly diagnosed or relapsed patients who achieved complete response (CR) or better after transplantation. In transplant-ineligible patients, experts recommended MRD testing in those who are ≤70 years old and in CR. If a patient was previously positive on positron-emission tomography and computed tomography (PET/CT), both MRD and PET/CT should be assessed at CR. MRD testing should be performed ≤6 months after transplantation and every 6–12 months in continuously treated patients in CR. There was no consensus on making treatment decisions based on MRD status. MRD testing is an important component of clinical management in MM. Additional data will further clarify the role of MRD in guiding treatment decisions.

## Graphical abstract

**Figure:**
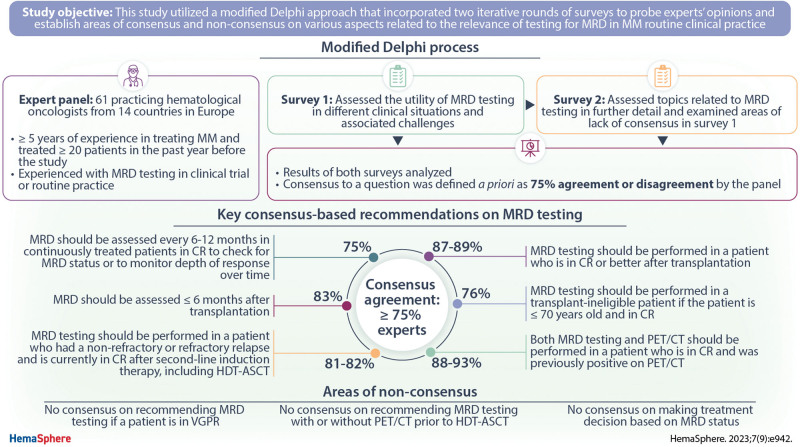

## BACKGROUND

Despite therapeutic advances in multiple myeloma (MM) that have improved survival outcomes,^1^ patients with MM who initially respond to therapy still face the risk of relapse. Relapse is often attributed to persistent chemotherapy-resistant cancer cells, including measurable residual disease (MRD), which remain undetectable using standard methods to evaluate treatment response.^2^

In recent years, results from multiple studies in newly diagnosed and relapsed/refractory MM indicated a prognostic value in determining MRD status; MRD negativity (defined as a threshold of sensitivity of <10^−4^, <10^−5^, or <10^−6^ tumor cells) was associated with improved survival outcomes, and in Programa para el Estudio de la Terapéutica en Hemopatías Malignas/Grupo Español de Mieloma (PETHEMA/GEM) trials, identified as a factor driving complete response (CR).^3–7^ A meta-analysis of 45 myeloma studies that included MRD analysis confirmed that MRD negativity was significantly associated with improved progression-free survival (PFS) and overall survival (OS).^8^ Several studies suggest that persistent MRD negativity could be an important efficacy marker.^9,10^ Data on improved survival outcomes with MRD negativity in MM are consistent with those from multiple other hematologic cancers.^11–13^

The observed benefits associated with MRD negativity have led the Foundation for the National Institute of Heath Biomarkers Consortium to examine the use of MRD in clinical trials and practice, the International Myeloma Working Group (IMWG) to include sustained MRD negativity as a response criterion in clinical trials, and regulatory agencies in Europe and the United States to accept MRD as an interim surrogate marker for PFS/OS.^14–19^ However, routine testing for MRD has not been fully integrated into MM management in the clinic.^15^ Contributing factors include insufficient clinical data on treatment decisions based on MRD status, lack of consensus on optimal sensitivity threshold for use of available MRD data to guide intervention, lack of comprehensive guidelines on testing in routine care, and difficulties with reimbursement.^15,20–22^ Technical challenges associated with MRD testing include identifying the most appropriate assay type (eg, next-generation flow [NGF] versus next-generation sequencing [NGS]), the need for skilled individuals to collect high-quality marrow samples for meaningful MRD analysis, and knowing when to measure extramedullary disease, which cannot be captured by marrow-based MRD assays, by positron-emission tomography (PET) for successful MM management.^6,15,23^ There are multiple studies designed to address these knowledge gaps. These include the recently published phase 2 studies of elotuzumab-based and daratumumab-based (Monoclonal Antibody-Based Sequential Therapy for Deep Remission in Multiple Myeloma [MASTER]) quadruplet combination treatments, which used MRD status to determine treatment duration and cessation.^24,25^ Furthermore, other clinical trials are ongoing for evaluating MRD-driven therapy in MM (eg, AURIGA, DRAMMATIC, and PERSEUS),^2,26^ and methods for detecting MRD in MM (MMRD; NCT02627261).

While the field awaits data from such clinical studies on MRD testing in MM, more timely and efficient but scientifically robust methods are needed to inform the practical utility of MRD testing in MM. Delphi and modified Delphi methods are established practices for collecting expert-based opinions on best practice care.^27^ Furthermore, these studies have the advantage of being able to address a wide range of questions in a timely manner that would not be possible with clinical studies. Delphi-based studies have proven applicability in informing clinical practice in different cancer types.^28–30^

This study used a modified Delphi approach to establish whether there was consensus on various aspects related to the relevance of testing for MRD in MM routine clinical practice to assess and confirm response to therapy. The results may inform MRD utility in MM management and design of future clinical studies.

## METHODS

### Study objectives

**Figure 1.**
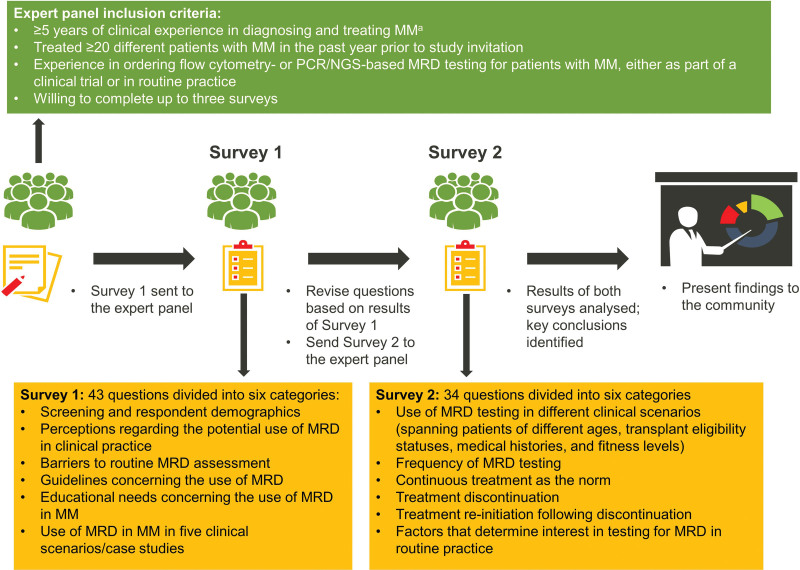
Modified Delphi process. ^a^Only clinicians with experience with MRD testing were invited to participate in the study, as the study was designed to assess their opinions on the use of MRD testing. MM = multiple myeloma; MRD = measurable residual disease; NGS = next-generation sequencing; PCR = polymerase chain reaction.

The objective of the study was to use a modified Delphi approach that incorporated up to a maximum of 3 iterative rounds of surveys (Figure 1) to probe expert panel members’ opinions to determine areas of consensus (agreement or disagreement) and nonconsensus concerning the value and utility of MRD testing in MM management, MRD testing in different clinical scenarios, and barriers to routine MRD assessment in the clinic.

### Delphi advisory committee and expert panel

An advisory committee of initially 12 experts in MM from across the Western Europe initially met on January 19, 2019. The advisory committee discussed the initiative and proposed and invited other suitable and internationally recognized MM hematological oncologists from their individual countries, currently involved in clinical investigation, to join them in forming a modified Delphi expert panel. The number of clinicians invited was based on the number of new MM cases in 2018 (Suppl. Table S1). One advisory committee member exited the study later due to a conflict of interests. Members of the expert panel were all confirmed by September 3, 2020.

### The modified Delphi process

During the initial face-to-face meeting, the advisory committee considered the methodology and developed and approved the questions for inclusion in survey 1, which addressed experts’ perceptions and challenges associated with MRD testing in different clinical scenarios (Figure 1). A link to the survey 1 questions was emailed to each expert panel member, and the responses collated by a third party uninvolved in assessing outcomes.

The pooled results of survey 1 were discussed virtually at a second advisory committee meeting on December 15, 2020. Based on these discussions, the advisory committee developed and approved a revised set of questions for survey 2 (Figure 1). Questions may have had refined wording or response options to probe topics in further detail or to clarify the original intentions of the survey 1 questions, or they may have been new questions integrating or addressing earlier categorical or free-text responses. Questions in survey 2 pertained mainly to areas of lack of consensus in survey 1. The process of distributing survey 2 to expert panel members, and collating and reviewing the responses, was the same as for survey 1. Survey 2 was completed on April 20, 2021.

Each of the 2 surveys was designed to be completed in ≈30 minutes; all responses were anonymous. The questions and response options are provided as online Supplemental Materials (Appendix 1 and 2).

Given that the COVID-19 pandemic occurred during the modified Delphi study, the study timeline was extended to ensure that the study did not interfere with the experts’ responsibilities in frontline clinical care.

### Data analysis and interpretation

When both survey results were available, the advisory committee reviewed the outcomes in a third virtual meeting and identified key conclusions. It was determined that a third survey round was not required, thus completing the study in April 2021.

### Statistics

The threshold level for consensus to an individual question in both surveys was defined *a priori* in the initial face-to-face advisory panel meeting as 75% (ie, agreement or disagreement of ≥46/61 respondents), which is within the range of consensus threshold for Delphi studies.^31^ Clustered consensus was defined as ≥75% agreement or disagreement for a general course of action related to a question in which clear consensus was not achieved for any one prespecified response option. General courses of action in each applicable question were determined by considering or clustering similar response options. All response data were summarized by descriptive statistics.

The modified Delphi study and the development of this report were driven by the advisory committee without external influence.

## RESULTS

### Expert panel characteristics

A total of 61 (11 advisors and 50 invited experts) hematological oncologists from 14 Western European countries made up the expert panel (Table 1; Suppl. Figure S1). Most members had 5–25 years of experience in treating MM (79%). All had experience in testing for MRD in MM (Table 1). All members completed survey 1 (Suppl. Table S2).

**Table 1:** Expert Panel Demographics (Survey 1)

	Expert Panel (N = 61; 11 Advisors, 50 Invited Experts)
Countries where members of expert panel were based, n	
Austria	2
Belgium	5
Finland	1
France	6
Germany	7
Greece	2
Ireland	1
Italy	8
Netherlands	5
Portugal	5
Spain	7
Sweden	2
Switzerland	2
United Kingdom	8
Years treating patients with MM, %	
<5	-
≥5–<25	79%
≥25–<50	19%
≥50	3%
Main clinical practice center, %	
Academic center/university teaching hospital	90%
Large private national institution	5%
Community hospital	3%
Other	2%
Number of patients with MM treated within the past 12 mo, %	
≥20–<30	5%
≥30–<40	3%
≥40–<50	5%
≥50	87%
Experience in using MRD testing in MM	
I use it only in clinical trials, when required by the study protocol	36%
I use it only in my own practice	3%
Both	61%

MM = multiple myeloma; MRD = measurable residual disease.

### Responses to survey 1

The following areas were considered in the first survey.

#### Perceptions of MRD testing in MM

Members of the expert panel achieved consensus agreement that MRD testing should be part of routine practice in MM (87%) and should be performed on bone marrow samples (95%; Table 2). They recommended (82%) that a 10^−5^ threshold be used as the minimum limit of MRD detection. Consensus agreement was not reached on the recommended frequency of MRD testing during the follow-up in general; however, 61% of respondents noted that this would depend on the patient’s response over time.

**Table 2:** Perceptions of MRD (Survey 1)

	Respondents (N = 61)
Should MRD testing become part of routine clinical practice in MM?	
▪ Yes	**87%**
▪ No	5%
▪ Maybe/I do not know	8%
When testing for MRD in patients with MM, of the following, what would you recommend as the minimum limit of detection?	
▪ 10^−4^	3%
▪ 10^−5^	**82%**
▪ 10^−6^	15%
In general, how frequently would you recommend testing for MRD in patients with MM in routine clinical practice?	
▪ One-off testing, as needed	2%
▪ Every 3 mo	5%
▪ Every 6 mo	10%
▪ Every year	18%
▪ No specific time point; depends on patient response over time	61%
▪ Other	5%^*a*^
What type of samples do you normally use to test MRD in your patients with MM in your routine clinical practice?	
▪ Peripheral blood	-
▪ Bone marrow	**95%**
▪ Both	5%

Percentages in bold denote consensus was reached.

^*a*^Response includes the following: “At key time points such as end of induction and post transplantation, and then every 6–12 months.”

MM = multiple myeloma; mo = months; MRD = measurable residual disease.

#### Barriers to determining MRD status in MM in routine practice

**Figure 2.**
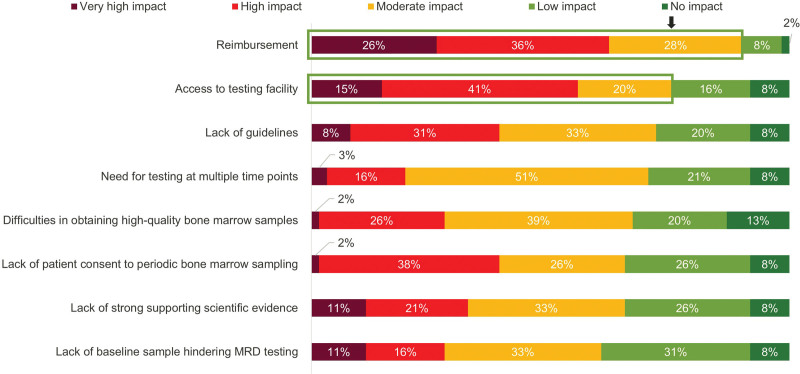
Barriers to determining MRD status in MM in routine clinical practice by the respondents (survey 1). N = 61 respondents. Ordered by moderate to very high impact. Arrow denotes 75% mark. Green boxes denote cluster consensus agreement (moderate to very high impact) was reached. MM = multiple myeloma; MRD = measurable residual disease.

While the experts reached consensus agreement that MRD testing should be implemented in routine clinical practice in MM, they identified the following 2 factors as having moderate to very high impact hindering clinicians in their home countries from performing MRD testing: reimbursement (90%) and access to a testing facility (76%; Figure 2).

#### Guidelines concerning the use of MRD

There was no consensus on which of the prognostic or treatment guidelines for MM are mostly used by the expert panel (Suppl. Table S3). Among the experts, the most commonly used source for prognostic guidelines was the IMWG (51%), and for treatment guidelines was the European Society for Medical Oncology (31%). Nearly half of the respondents (48%) confirmed that MRD was not covered in the prognostic or treatment guidelines that they used.

#### Educational needs concerning the use of MRD in MM

The experts reached consensus agreement (84%) that clinicians in their home country were not suitably informed regarding MRD testing in MM (Suppl. Table S4). The experts also reached consensus agreement that they (79%) or the clinicians in their home countries (92%) would benefit from additional education on the use of MRD in informing treatment decisions. While most experts (61%) agreed that they themselves would benefit from guidelines on the use of MRD in MM, consensus was reached (75%) that clinicians in their home countries would benefit from having such guidelines.

#### Use of MRD in MM (5 case studies)

The 5 case descriptions and experts’ answers to the questions are provided in Appendix 3. These case scenarios allowed evaluation of patient characteristics that would impact the decision to test for MRD and the treatment decision based on MRD status.

The first case described a treated and transplant-eligible patient who was positive on PET/computed tomography (CT) before transplant, in CR, and was awaiting high-dose therapy and autologous stem cell transplant (HDT-ASCT). Experts reached consensus agreement that they would recommend MRD testing if a patient had undergone HDT-ASCT and was in CR (82%). Knowing MRD status is not critical before transplant, most experts (89%) would therefore proceed to transplant regardless of MRD status. The frequency of MRD assessment was unclear; a combined 85% of experts would assess MRD ≤3 months after transplantation (74% would test at 3 months and 11% in <3 months), and a combined 76% of experts would assess MRD every ≥3 months during maintenance therapy after transplant (38% annually, 33% every 6 months, and 5% every 3 months). If a patient was positive on PET/CT, then the experts agreed that they would perform both MRD testing and PET/CT after HDT-ASCT, as these tests would provide complementary information (75%–85%).

The second and third cases described a transplant-ineligible patient in CR and a transplant-eligible patient in very good partial response (VGPR), respectively. The fourth case described a patient who had relapsed after transplantation; the patient subsequently achieved CR with continuous treatment. The results from these 3 clinical scenarios showed that, due to different expert opinions, the experts did not reach consensus on recommending MRD testing if a patient was transplant ineligible, in VGPR, or had a relapse after prior transplantation. Furthermore, the experts did not agree on the decision to discontinue treatment if a patient was MRD negative; however, there was consensus to continue treatment if the patient was MRD positive (≈80%). Finally, if the experts had decided to proceed with MRD testing, there was no consensus on the frequency of testing to check for MRD status.

The fifth case described a posttransplant patient in CR who wanted to cease treatment (reason not specified in the survey). This scenario described patient characteristics that would impact the experts’ decision to test for MRD. The experts reached consensus agreement that they would support MRD testing if the patient was <70 years old (88%–91%), newly diagnosed (90%), transplant-eligible (84%), on first-line therapy (97%), classified as having high-risk genetics with or without a stage III determination using the Revised International Staging System ([R-ISS]; 86%–91%), or had a first relapse (88%).

Areas of consensus and nonconsensus regarding MRD testing are summarized in Table 3.

**Table 3:** Key Consensus-based Recommendations on MRD Testing and Areas of Nonconsensus From the 5 Case Studies in Survey 1

**Key consensus-based recommendations**
▪ MRD testing should be performed in a patient who had received HDT-ASCT and is in CR
▪ MRD testing should be performed ≤3 mo after transplantation
▪ Both MRD testing and PET/CT should be performed after HDT-ASCT in a patient who is in CR and was previously positive on PET/CT
**Areas of nonconsensus**
▪ There was no consensus on recommending MRD testing ○ If a patient is transplant ineligible ○ If a patient is in VGPR ○ If a patient has a relapse after prior transplantation
▪ There was no consensus on recommending treatment discontinuation if a patient is MRD negative
▪ There was no consensus on frequency of MRD testing should test be performed

CR = complete response; HDT-ASCT = high-dose therapy and autologous stem cell transplant; mo = months; MRD = measurable residual disease; PET/CT = positron-emission tomography/computed tomography; VGPR = very good partial response.

### Responses to survey 2

Survey 2 was developed to further understand the panel’s responses in the 5 case studies in survey 1 (Appendix 3). The specific themes addressed are the use of MRD testing in different clinical scenarios, frequency of MRD testing, treatment discontinuation, treatment reinitiation, and patient- and disease-related factors driving MRD testing. Of the 61-member expert panel, 53 members (87%), including all 11 advisors, completed survey 2 (Suppl. Table S2).

#### Use of MRD testing in different clinical scenarios (spanning patients of different ages, transplant eligibility statuses, medical histories, and fitness levels)

Descriptions of the 16 clinical scenarios in survey 2 and experts’ answers to the questions are provided in Appendix 4.

The 16 scenarios in Survey 2 re-evaluated areas of consensus and nonconsensus in the 5 case studies in survey 1. Results from these clinical situations showed that the experts would recommend MRD testing in a posttransplant patient who was in CR or better (87%–89%); however, there was no consensus if the patient was in VGPR. If the patient in CR was previously positive on PET/CT, the experts reached consensus agreement that they would perform both MRD testing and PET/CT after transplantation, because the information from both tests is complementary (88%–93%). While the experts would likely recommend MRD testing and PET/CT before HDT-ASCT in the same scenario (87%), there was no consensus on whether the tests would provide meaningful information. If a patient was transplant-ineligible and in CR while on maintenance treatment, the experts would recommend performing MRD testing if the patient was ≤70 years old (76% agreement); there was no consensus about MRD testing if the patient was ≥75 years old with the assumption that they were willing to be tested and that there were no logistical or technical barriers to testing. However, the experts believed in general that MRD testing is useful in certain or most instances if the patient was fit and in CR regardless of age (81%–91% agreement); consensus agreement was also reached that MRD testing is not useful if the patient was frail and not in CR (79%–89%). The final scenario described a patient achieving CR with treatment plus HDT-ASCT after relapse. In this situation, ≈82% of experts would recommend performing MRD testing; however, the experts did not reach consensus on MRD testing if the patient had not received HDT-ASCT.

#### Frequency of MRD testing (after transplantation and during continuous treatment)

The frequency of MRD testing after transplant was probed in case study 1 of survey 1, but there was no clear consensus on how regularly MRD testing should be performed (Appendix 3). The issue of testing frequency was re-examined in survey 2 (Suppl. Table S5). Results showed that in a patient who had undergone transplant, a combined 83% of experts agreed they would wait up to 6 months (57% would wait ≤3 months and 26% would wait 4–6 months) before testing for MRD. If a patient was on continuous maintenance therapy, after achieving CR with or without ASCT, then 75% of experts agreed that sustained MRD status or attainment of MRD status should be confirmed every 6 months to a year. In a patient who was in CR, had undergone ASCT, and was MRD negative during 2 years of maintenance therapy, a combined 94% of experts agreed that MRD testing should occur at intervals of longer than 6 months.

#### Treatment discontinuation

For the patient described in case study 1 of survey 1, 74% of experts (no consensus) would support therapy discontinuation if the patient was in CR with 2 years of negative status on MRD and PET/CT (Appendix 3). The scenario in survey 2 described a patient who was in CR and negative for MRD and on PET/CT for 2 years or more. The patient expressed a desire to discontinue treatment. While consensus was not reached, 70% of respondents would support treatment discontinuation in this case. If a patient has ceased therapy, 87% of experts agreed that long-term MRD testing is important (Suppl. Table S6).

#### Treatment re-initiation following discontinuation

For the patient who discontinued therapy described in case study 1 of survey 1, experts did not reach a consensus agreement on re-initiating therapy or the use of MRD status to drive the therapeutic decision (Appendix 3). The survey 2 scenario described a patient who was MRD negative and discontinued treatment, but relapsed. Experts did not reach a consensus agreement on the type of relapse—biochemical, clinical, or MRD positivity—that would prompt them to reinitiate treatment (Suppl. Table S7).

#### Patient- and disease-related factors that determine interest in testing for MRD

**Figure 3.**
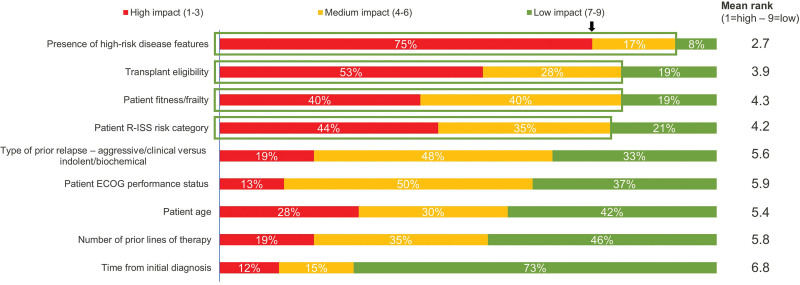
Factors that determine interest in testing for MRD (survey 2). N = 53 respondents. Ordered by medium to high impact. Arrow denotes 75% mark. Green box denotes cluster consensus was reached that the factors have medium to high impact on decision to test for MRD. ECOG = Eastern Cooperative Oncology Group; MRD = measurable residual disease; R-ISS = Revised International Staging System.

In case study 5 of survey 1, the majority of experts stated that they would either consider various patient or disease characteristics in making a decision for MRD testing or that they would be inclined to test for MRD regardless of patient or disease characteristics (Appendix 3). In survey 2, respondents reached consensus agreement that the following factors have medium to high impact on their decision to test for MRD: (1) presence of high-risk disease features (no specific definition provided in survey 2; 92%); (2) transplant eligibility (81%); (3) patient risk category using R-ISS (79%); and (4) patient fitness/frailty (80%; Figure 3).

Areas of consensus and nonconsensus regarding MRD testing based on survey 2 results are summarized in Table 4.

**Table 4:** Key Consensus-based Recommendations on MRD Testing and Areas of Nonconsensus Based on the Clinical Situations in Survey 2

**Key consensus-based recommendations on MRD testing**
▪ MRD testing should be performed in a patient who is in CR or better after transplantation
▪ MRD testing should be performed in a transplant-ineligible patient if the patient is ≤70 years old and in CR^*a*^
▪ Both MRD testing and PET/CT should be performed in a patient who is in CR and was previously positive on PET/CT
▪ MRD testing should be performed in a patient who had a nonrefractory or refractory relapse and is currently in CR after second-line induction therapy, including HDT-ASCT
▪ MRD should be assessed ≤6 mo after transplantation
▪ MRD should be assessed every 6–12 mo in continuously treated patients in CR to check for MRD status or to monitor depth of response over time
**Areas of nonconsensus**
▪ There was no consensus on recommending: ○ MRD testing if a patient is in VGPR ○ MRD testing with or without PET/CT before HDT-ASCT
▪ There was no consensus on making treatment decision based on MRD status

^*a*^Experts’ general view is that MRD testing is useful in patients who are fit and in CR regardless of age.

CR = complete response; HDT-ASCT = high-dose therapy and autologous stem cell transplant; mo = months; MRD = measurable residual disease; PET/CT = positron-emission tomography/computed tomography; VGPR = very good partial response.

## DISCUSSION

MRD testing is not widespread in routine clinical practice for reasons such as insufficient MRD-testing guidelines, availability, and budget. Therefore, there are multiple ongoing trials examining the use of MRD testing in MM and MRD-directed treatments to enhance utilization as an intervention-directing biomarker.^2,26^ To bridge the knowledge gap, we invited 50 other experts from Europe to participate in our modified Delphi study to understand the use of MRD in clinical practice for patients with MM. We identified areas of consensus agreement on MRD testing in clinical practice that were based on available clinical data and areas of nonconsensus; these findings require additional clinical studies to confirm or resolve. Unlike some other Delphi studies in blood cancers, the current study employed a rigorous methodology involving 2 rounds of surveys answered by a large panel of experts, including 90% who work in academic settings and 36% who use MRD testing only in clinical trials, to identify and confirm areas of consensus and nonconsensus regarding MRD testing in a variety of clinical situations.^32–34^

In this modified Delphi study, the majority of experts (87%) agreed that MRD should be assessed in patients with MM in routine clinical practice, especially in patients who achieved CR or better (75%–89%) after completing ASCT because the PETHMA/GEM trials identified MRD as a reliable predictor for OS.^4^ They agreed (82%–95%) that MRD should be assessed in the bone marrow and that the assay’s minimum threshold of detection should be 10^–5^. These recommendations are based on the clinical data and aligned with prior guidance.^5,7,35,36^

The experts reached consensus agreement that a patient’s transplant eligibility, fitness level, and presence of high-risk disease features are key factors driving the decision to test for MRD. Consensus-based recommendations for MRD testing in different clinical situations are as follows:

- MRD should be determined if a patient is fit, transplant-eligible, and has achieved CR (expert agreement: 81%–97%). This recommendation is consistent with published data on the prognostic value of MRD negativity in patients who were newly diagnosed with MM.^5,7,36^
- PET/CT and MRD testing are complementary tests for determining MRD after transplantation in a transplant-eligible patient who is positive on PET/CT at the time of diagnosis and in CR after ASCT (expert agreement: 88%–93%). This determination aligns with the results from a clinical study and the IMWG’s conclusion that patients’ disease outcomes improve when they are negative on PET/CT and for MRD in the bone marrow, compared with being negative on either measurement alone.^17,37^ Furthermore, it aligns with IMWG’s recommendation to use PET/CT to track changes in positive lesions in the setting of MRD negativity in the marrow.^17^
- If a transplant-eligible patient relapses after responding to the first-line therapy, MRD should be assessed after the patient has received a second induction plus transplantation and is in CR while on maintenance therapy (expert agreement: 81%–82%). This clinical scenario is less likely due to use of potent combination therapy at relapse. But recent trials demonstrate that a high CR rate is associated with MRD negativity in relapsed MM.^38,39^ The preference for MRD testing after transplantation is based on the observation that patients in the IFM 2009 study who received transplantation had significantly higher response rates versus those who did not.^40^ Furthermore, transplantation reduced the risk of disease progression or death in the DETERMINATION trial.^41^ These Delphi results and supporting evidence support MRD testing after first transplantation. The utility of MRD testing after second transplantation was not evaluated in this study due to lack of data.
- If a patient is transplant-ineligible and in CR while on maintenance treatment, MRD should be assessed if the patient is ≤70 years (expert agreement: 76%). However, the experts did not reach consensus on testing for MRD in patients aged ≥75 years in a similar scenario, likely due to infrequent sampling of marrow in this age group in clinical practice. While the patient’s age and clinical situation are factors in experts’ MRD-testing decision-making, the experts’ general view based on their clinical experience is that MRD testing is useful in patients who are fit and in CR regardless of age, including those who are ≥75 years old and willing to be tested (expert agreement: 81%–91%). These recommendations accounting for age, CR, and transplant-ineligible status of patients are supported by results of the MAIA and ALCYONE trials, which enrolled patients with similar criteria. These trials reported that up to a third of patients achieved MRD negativity including a subset with sustained negativity status.^42^ Higher MRD-negativity rates were associated with prolonged OS and PFS in transplant-ineligible patients regardless of age.^43,44^ Sustained MRD negativity is one of the treatment goals pursued by cooperative groups as a biomarker during maintenance and for considering treatment discontinuation for myeloma patients regardless of transplantation status.^19,26^ Because bone marrow aspiration is required to confirm CR status, simultaneous MRD assessment of the marrow would provide meaningful clinical information if the MRD-testing technology is available to the clinician.
- MRD testing is recommended if a patient exhibits high-risk disease features (expert agreement: 92%). Furthermore, if a patient wishes to discontinue treatment, MRD status and high-risk genetics or high-risk plus R-ISS III categories are key factors in deciding whether or not to support their wish (expert agreement: 86%–91%). This decision reflects findings from analyses of multiple trials including POLLUX, CASTOR, ALCYONE, MAIA, and Myeloma XI, which demonstrated an association between improved PFS and low/standard versus high cytogenetic risk status;^25,45,46^ the PETHEMA/GEM2012MENOS65 study in which patients with high-risk disease who achieved MRD negativity had improved disease outcomes as those with standard-risk disease;^47^ the MASTER trial, which showed increased incidence of disease progression and MRD resurgence following treatment cessation in patients who had ≥2 compared with those with ≤1 high-risk cytogenetic abnormalities;^24^ and the OPTIMUM study in which extended and intensified post-ASCT consolidation treatment was associated with sustained MRD negativity in patients with ultra-high-risk disease features.^48^ In practice, patients with high-risk disease are often treated at biochemical relapse before clinical symptoms emerge. The panel findings are a validation of this approach, but adhere to a much lower threshold of MRD positivity in the marrow.
- MRD should be assessed within 6 months after transplantation. The question of when to determine MRD after transplantation was evaluated in both surveys (expert agreement: 85% for ≤3 months in survey 1 and 83% for ≤6 months in survey 2). This determination is based on experts’ clinical experience; the 3–6 month timeframe allows recovery of immune functions after transplantation. MRD testing should be repeated every 6 months to a year if a patient is on continuous treatment and in CR (expert agreement: 75%). This determination is supported by a study that assessed MRD once a year for up to 5 years to examine the long-term benefit of MRD negativity during maintenance treatment with lenalidomide;^49^ furthermore, it aligns with standard practice in the management of acute lymphoblastic leukemia as regular testing is to determine sustained MRD negativity.^50^ More studies are needed to establish testing frequency in different clinical scenarios in MM.

This Delphi study also identified areas in which the experts did not reach consensus agreement. A key area of nonconsensus was whether the expert panel would support treatment discontinuation in a patient who was in CR and had been negative for MRD on PET/CT for 2 years or more. This may be partly due to a patchy pattern of tumor cell distribution in the bone marrow, which may lead to ambiguity in MRD-negativity results.^51^ Furthermore, there was a lack of data on supporting treatment discontinuation based on MRD negative status at the time of this Delphi study. However, subsequent data from the GEM2014MAIN study revealed a favorable PFS rate over time in patients who were MRD negative after 2 years of maintenance treatment and discontinued therapy versus those who were MRD positive and remained on therapy.^52^ A recently published report on the MASTER trial showed that of the newly diagnosed patients with MM plus ≤1 high-risk cytogenetic abnormalities who ceased treatment because of 2 consecutive MRD-negative assessments, the risk of progression or resurgence of MRD was low (4%) after 12 months, suggesting that discontinuing treatment in such patients may be feasible in the real world.^24^ Another ongoing prospective trial RADAR in the United Kingdom may further shed light on the feasibility of treatment discontinuation in patients newly diagnosed with MM who achieved MRD negativity.^53^ In the IFM 2009 study, investigators suggested that transplantation may be avoided in MRD-negative patients in the transplant or nontransplant study groups who received maintenance therapy for a year given a lack of difference in OS between the 2 groups.^40^ This finding provides a scenario for considering only fixed duration maintenance therapy without transplantation in MRD-negative patients. Another area of nonconsensus was the type of relapse (biochemical or MRD positive) in patients who discontinued treatment that would prompt treatment reinitiation. This is an area of active investigation and debate; however, studies suggest treatment at biochemical relapse has resulted in an overall response rate of 36%–82%.^54–56^ As shown in the ENDEAVOR trial, the variability in the response was driven in part by the treatment (carfilzomib+dexamethasone or bortezomib+dexamethasone) following a relapse and when the treatment was initiated (biochemical versus clinical relapse).^56^ A third area of nonconsensus was whether to perform MRD testing in patients who achieved VGPR, although the GEM2012MENOS65 study showed similar survival outcome in patients who were MRD negative and in CR or better, or VGPR.^57^ The overall results suggest that the decision to assess for MRD in VGPR is more complex, which depended on factors such as the definition of VGPR, transplantation status, bone marrow plasma cell percentage, and patient fitness status. In general, the lack of agreement is due to insufficient data, therefore expert judgements were based on individual experiences, philosophy, and standard practices at different centers.

The experts identified 3 challenges to MRD testing. First, the experts concluded that clinicians are not suitably informed regarding the use of MRD testing in MM (expert agreement: 79%–92%) and therefore more education is needed. The second challenge pertains to access to testing (expert agreement: 76%). While NGF-based testing is widely available, there is not a uniform standard for MRD testing.^18^ For NGS-based testing, the technology is not widely available, and is costly to set up at medical centers, requiring initial equipment investment and a specialized workforce.^58^ Third, the experts raised concerns that medical facilities may encounter difficulties in reimbursement from payers for performing MRD testing (expert agreement: 90%). This sentiment was echoed in another Delphi study on the use of NGS that concluded that payer coverage is variable and inconsistent due to policy differences in assessing the clinical utility of NGS.^59^ To overcome these challenges, a key first step is to improve scientific knowledge, which is being addressed with ongoing and future clinical studies on MRD testing and MRD-directed therapy in MM. When the results from these studies become available, comprehensive guidelines can be developed to inform clinicians on MRD-directed treatment approaches and timing and frequency of MRD testing. The guidelines may also guide payers on developing uniform policies for MRD testing for consistent reimbursement for the procedure. Arguably, the cost of MRD testing will be more acceptable if MRD-directed therapies lower the cost of a patient’s overall treatment plan. A study in Germany developed a health-economic model, based mainly on the evidence from the IFM 2009 study, to evaluate the economic impact of MRD testing. The study estimated that using NGS may save €18,396 in 1 year, €69,991 after 3 years, and €77,140 after 10 years per patient for treatment in those who are tested for MRD versus those not tested, because healthcare providers can avoid unnecessary and costly MM treatment if MRD status is known.^60^ A streamlined process of MRD-directed therapy that would likely be covered by payers may incentivize hospitals to invest in upgrading their testing facilities.

This Delphi study was limited in its evaluation of MRD testing in a general context of patient factors (eg, transplant eligibility and fitness), and standard MM treatments (including daratumumab, bortezomib, lenalidomide, dexamethasone, and ASCT) and responses to those therapies. Examination of more specific factors such as differences in treatment and testing access by country and the impact of individual MM drugs were beyond the scope of this study. The study design circumvented any real-world barriers concerning access to treatment and MRD testing with the assumptions that such barriers did not exist. While certain treatment combinations were more effective than others in managing MM as shown in phase 3 trials, PFS was determined by patients who achieved CR or better and MRD negativity, regardless of therapy,^41,45^ supporting the notion that patient factors and responses were key to MRD-testing decision-making rather than the type of treatments the patients received.

## CONCLUSIONS

The lack of comprehensive guidelines and education on MRD testing has hindered its use in MM management in the real world. This modified Delphi study has established the relevance of MRD testing in the clinic and provided consensus-based suggestions on its use in different clinical scenarios. The consensus agreements were supported by published data, including those published after the completion of the study. Until additional data from clinical studies become available, results from this study may inform clinicians in the use of MRD testing in MM management.

## Acknowledgments

Writing and editorial support for the development of this publication was provided by Ying Jean, PhD and Delisa O’Brien of Ashfield MedComms (New York, NY), an Inizio Company; funding was provided by the study sponsor. The authors had full control of the publication and provided their final approval of all content. The Delphi meetings were facilitated by Leon Newman, PhD, and Kindiya Geghman, PhD, of Ashfield MedComms, and funded by the study sponsor. The authors would like to thank the following experts who participated as members of the expert panel in this modified Delphi study: Heinz Ludwig from Austria; Chantal Doyen, Isabelle Vandebroek, Marie-Christiane Vekemans, and Nathalie Mueleman from Belgium; Raija Silvennoinen from Finland; Aurore Perrot, Florent Malard, and Alexis Talbot from France; Hartmut Goldschmidt, Cyrus Khandanpour, Christoph Scheid, Annamaria Brioli, Markus Munder, Hermann Einsele, Raphael Teipel from Germany; Meletios A. (Thanos) Dimopoulos from Greece; Vitaliy Mykytiv from Ireland; Niccolò Bolli, Stefania Olivamolinet, Pellegrino Musto, Massimo Offidani, Elena Zamagni, Daniele Derudas from Italy; Mark David Levin, Wilfried Roeloffzen, Annemeik Broijl, Inger Nijhof from the Netherlands; Tobias Slördahl from Norway; Catarina Geraldes, Paulo Lucio, Rui Bergantim, Graça Vasconcelos Esteves, and Ana Maria Lopez de Macedo from Portugal; Enrique Colado, Marta Sonia Gonzalez Perez, Javier De la Rubia, Rafael Ríos Tamayo, and Maria Jesus Blanchard from Spain; Markus Hansson from Sweden; Christoph Driessen and Christoph Renner from Switzerland; Sally Moore, Kwee Yong, Matthew Jenner, Guy Pratt, Reuben Benjamin, Supratik Basu, and Rachel Hall from the United Kingdom.

## Author contributions

KR, HA-L, CHB, MD, FG, SM, JM-L, MVM, MM, NWCJvdD, and KW (members of the advisory committee) did conceptualization, methodology, formal analysis, writing-review, and editing.

## Disclosures

KR: Has served as an advisor for and received honoraria from Janssen, Adaptive Biotechnologies, Amgen, Takeda, Abbvie, Oncopeptides, Celgene – Bristol Myers Squibb, Pfizer, and GSK. Has received grant funding from Janssen, Amgen, Takeda, GSK and Celgene – Bristol Myers Squibb. HA-L: Has served as an advisor for and received honoraria from Amgen, Bristol Myers Squibb, Celgene, Janssen, Sanofi, GSK, Takeda, and Adaptive Biotechnologies. CHB: Has served as an advisor for and received honoraria from Amgen, Bristol Myers Squibb, Takeda, Sanofi, GSK, and Adaptive Biotechnologies. MD: Has served as an advisor for and received honoraria from Amgen, Celgene – Bristol Myers Squibb, GSK, Janssen, Sanofi, and Takeda. FG: Has served as an advisor for Janssen, Amgen, Celgene – Bristol Myers Squibb, Adaptive Biotechnologies, Roche, Abbvie, GSK, Takeda, Bluebird Bio, Oncopeptides, Pfizer, and Sanofi. Has received honoraria from Janssen, Amgen, Celgene – Bristol Myers Squibb, GSK, Takeda, and Sanofi. SM: Has served as an advisor for Abbvie, Adaptive Biotechnologies, Amgen, Celgene – Bristol Myers Squibb, GSK, Janssen, Oncopeptides, Pfizer, Regeneron, Roche, Sanofi, and Takeda. JM-L: Has served as an advisor for and received honoraria from Amgen, Bristol Myers Squibb, Celgene, Janssen, Sanofi, GSK, Incyte, Roche, Pfizer, Novartis and Adaptive Biotechnologies. MVM: Has served as an advisor for and received honoraria from Janssen, Celgene – Bristol Myers Squibb, Takeda, Amgen, Adaptive Biotechnologies, Oncopeptides, Sanofi, Roche, Regeneron, and Pfizer. MM: Has received honoraria from Amgen, Astellas, Bristol Myers Squibb, Celgene, Gilead, Janssen, Jazz, Takeda, Novartis, Pfizer, Sanofi, and Adaptive Biotechnologies. Has received research funding from Celgene, Janssen, Jazz, and Sanofi. NWCJvdD: Has served as an advisor for Janssen Pharmaceuticals, Amgen, Adaptive Biotechnologies, Celgene, Bristol Myers Squibb, Novartis, Roche, Takeda, Bayer, and Servier. Has received grant funding from Janssen Pharmaceuticals, Amgen, Celgene, Cellectis, and Bristol Myers Squibb. KW: Has served as an advisor for Amgen, Adaptive Biotechnologies, Bristol Myers Squibb, Celgene, GSK, Janssen, Karyopharm, Oncopeptides, Sanofi, and Takeda. Has received honoraria from Amgen, Adaptive Biotechnologies, Bristol Myers Squibb, Celgene, GSK, Janssen, Karyopharm, Oncopeptides, Sanofi, Takeda, and Roche. Has received grant funding from Amgen, Janssen, Celgene, and Sanofi (to the institution).

## Sources of funding

The research for this study was sponsored by Adaptive Biotechnologies (Seattle, WA).

## Supplementary Material


