# Correction Notice: Measurable Residual Disease Testing in Multiple Myeloma Routine Clinical Practice: A Modified Delphi Study

**DOI:** 10.1097/HS9.0000000000000986

**Published:** 2023-11-02

Since the publication of the article entitled “Measurable Residual Disease Testing in Multiple Myeloma Routine Clinical Practice: A Modified Delphi Study” (*HemaSphere.* 2023;7(9):e942), a correction is needed for Table 3 and Table 4. Bullet points and subheaders were misaligned, therefore reducing clarity for readers. This has now been adjusted. The journal office apologizes for any inconvenience.

The changes have been made online: https://journals.lww.com/hemasphere/fulltext/2023/09000/measurable_residual_disease_testing_in_multiple.12.aspx

