# Supplementary material for: Measurable Residual Disease Testing in Multiple Myeloma Routine Clinical Practice: A Modified Delphi Study
Source: Hemasphere. 2023 Aug 30;7(9):e942. doi: 10.1097/HS9.0000000000000942 (PMC10470794; doi:10.1097/HS9.0000000000000942)
Supplement: Supplementary file 1 [file hs9-7-e942-s001.pdf]

Supplemental Digital Content

Measurable residual disease testing in multiple myeloma routine clinical practice: a modified Delphi study

Karthik Ramasamy[1], Hervé Avet-Loiseau[2], Cecilie Hveding Blimark[3], Michel Delforge[4], Francesca Gay[5], Salomon Manier[6], Joaquín Martinez-Lopez[7], Maria Victoria Mateos[8], Mohamad Mohty[9], Niels W.C.J. van de Donk[10], Katja Weisel[11]

[1]Oxford University Hospitals NHS Foundation Trust, Radcliffe Department of Medicine, Oxford University, UK
[2]University Institute of Cancer Toulouse, University Hospital of Toulouse, Toulouse, France
[3]Sahlgrenska University Hospital, Gothenburg, Sweden
[4]University Hospital Leuven, Leuven, Belgium
[5]University of Torino, Turin, Italy
[6]University of Lille, Lille, France
[7]12 de Octubre University Hospital, Madrid, Spain
[8]University Hospital of Salamanca, Salamanca Biomedical Research Institute (IBSAL), CIC, Ciberonc, Salamanca, Spain
[9]Hospital Saint-Antoine, Sorbonne University, INSERM UMRs 938, Paris, France
[10]VU University Medical Center, Amsterdam, Netherlands
[11]University Medical Center Hamburg-Eppendorf, Hamburg, Germany

Table of contents

Section	Page
Supplementary tables and figures	2
Appendix 1: Survey 1 questions	12
Appendix 2: Survey 2 questions	37
Appendix 3: Survey 1 case studies and results	65
Appendix 4: Survey 2 scenarios and results	80

Supplementary Table S1. Incidence and mortality rates of MM in Western European countries in 2018[a,b]

Country	Population	Number of new cases of MM in 2018	Incidence rate per 100,000 (age standardized) 2018	Mortality rate per 100,000 (age standardized) 2018
Germany	84M	7,131	7.6	4.8
UK	68M	6,757	10.6	5.2
France	65M	6,205	9.3	4.9
Italy	60M	6,034	8.6	5
Spain	47M	3,261	6.7	4.2
Netherlands	17M	1,185	7	6.1
Portugal	10M	1,034	9	6.1
Belgium	11M	1,029	9.2	5
Sweden	10M	818	8.2	5.5
Greece	10M	809	6.8	4.1
Switzerland	9M	673	8	4.9
Austria	9M	568	6.2	4.9
Norway	5M	475	10.1	6.7
Finland	6M	471	8	4.8
Denmark	6M	374	6.5	5.4
Ireland	5M	325	9	6

[a]European countries from which experts were selected had similar age-standardized rates of MM incidence (6.7–10.6/100,000) and MM associated mortality (4.1–6.0/100,000) in 2018. The number of clinicians invited per country was based nominally on the number of new MM cases in 2018: five clinicians were invited from countries with >3000 new MM cases, three from countries with ≥1000–3000 new MM cases, and two from countries with <1000 new MM cases

[b]Table was created based on incidence and mortality estimates provided by:

- Cancer Today (World Health Organization – International Agency for Research on Cancer)
    - Website: https://gco.iarc.fr/today/home
    - Given that 2020 data are now released, 2018 data are no longer available
- European Cancer Information System (European Commission)[1]
    - Website: https://ecis.jrc.ec.europa.eu/

MM, multiple myeloma; M, million

Reference

1. Bettio M, Negrao De Carvalho R, Dimitrova N, et al. Dataset collection: European Cancer Information System, European Commission, 2018, JRC111427.

Supplementary Table S2. Targeted and actual completion of Surveys 1 and 2 by country

Country	Target number (range) of respondents by country	Recruiting advisor[a]	Completed Survey 1, n	Completed Survey 2, n
Austria	2 (1–4)	Salomon Manier	2	1
Belgium	3 (2–6)	Michel Delforge	5	5
Finland	2 (1–4)	Cecilie Hveding Blimark	1	-
France	5 (4–10)	Hervé Avet-Loiseau & Mohamad Mohty	6	6
Germany	5 (4–12)	Katja Weisel	7	5
Greece	2 (1–4)	Hervé Avet-Loiseau	2	2
Ireland	2 (1–4)	—[b]	1	1
Italy	5 (4–10)	Francesca Gay	8	6
Netherlands	3 (2–6)	Niels W.C.J. van de Donk	5	4
Portugal	3 (2–6)	Maria Victoria Mateos	5	5
Spain	5 (4–8)	Maria Victoria Mateos & Joaquín Martinez-Lopez	7	7
Sweden	2 (1–4)	Cecilie Hveding Blimark	2	2
Switzerland	2 (1–4)	Michel Delforge	2	1
United Kingdom	5 (4–10)	Karthik Ramasamy	8	8
Total	50 (34–100) Overall target minimum: 42		61	53

[a]All 11 advisors completed Surveys 1 and 2, and are included in the final counts

[b]Advisor discontinued participation after recommending an expert from Ireland to participate in the study

Supplementary Table S3. Prognostic or treatment guidelines used (Survey 1)

	Respondents (N=61)
**Name(s) of the prognostic guidelines used**	
▪ IMWG	51%
▪ R-ISS	20%
▪ EMN	11%
▪ ISS	7%
▪ ESMO	3%
▪ Onkopedia	3%
**Names(s) of treatment guidelines used**	
▪ ESMO	31%
▪ IMWG	25%
▪ EMN	18%
▪ IFM	10%
▪ Onkopedia	7%
▪ NICE	7%
**IS MRD covered in the prognostic or treatment guidelines that you use?**	
▪ MRD is covered in the prognostic guidelines that I use	34%
▪ MRD is covered in the treatment guidelines that I use	3%
▪ MRD is covered in both the prognostic and treatment guidelines I use	13%
▪ MRD is not covered in the prognostic or treatment guidelines I use	48%
▪ I do not use guidelines	-
▪ I'm not sure	2%

EMN, European Myeloma Network; ESMO, European Society for Medical Oncology; IMWG, International Myeloma Working Group; IFM, Intergroupe Francophone du Myelome; ISS, International Staging System; MM, multiple myeloma; MRD, measurable residual disease; NICE, National Institute for Health and Care Excellence; R-ISS; Revised Multiple Myeloma International Staging System

Supplementary Table S4. Educational needs regarding the determining MRD status in MM (Survey 1)

	Respondents (N=61)
**Do you feel that you are suitably informed regarding the use of MRD in MM such that you can use it with confidence in a variety of clinical situations?**	
▪ Yes	64%
▪ No	36%
**Do you feel that clinicians in your country are suitably informed regarding the use of MRD in MM such that they can use it with confidence in a variety of clinical situations?**	
▪ Yes	16%
▪ No	**84%**
**In which of the following categories do you feel that you or clinicians in your country would benefit from more education/resources regarding MRD in MM? Please select all that apply.**	
▪ Methodologies available for testing MRD	
o Respondent	31%
o Other clinicians in respondent's country	57%
▪ Use of MRD in prognostication	
o Respondent	28%
o Other clinicians in respondent's country	64%
▪ Use of MRD in informing treatment decisions	
o Respondent	**79%**
o Other clinicians in respondent's country	**92%**
▪ Guidelines on the use of MRD in MM	
o Respondent	61%
o Other clinicians in respondent's country	**75%**
▪ Other	
o Respondent	16%
o Other clinicians in respondent's country	5%

Percentages in bold denote consensus was reached

MM, multiple myeloma; MRD, measurable residual disease

Supplementary Table S5. Frequency of MRD testing (after transplantation and during continuous treatment; Survey 2)

	Respondents (N=53)
**A patient has undergone ASCT, how long would you typically recommend waiting post-ASCT before taking a fresh bone marrow sample for MRD assessment?**	
▪ ≤3 months	57%[a]
▪ 4–6 months	26%[a]
▪ >6 months	11%
▪ I am not sure	6%
**What is the minimum frequency at which you would recommend testing for sustained MRD status in bone marrow in a patient who has undergone ASCT, is in CR, and has recently (within the past year) been initiated on maintenance therapy?**	
▪ ≤6 monthly	
o MRD- patient	8%
o MRD+ patient	23%[b]
▪ >6 monthly to annually	
o MRD- patient	**75%**
o MRD+ patient	64%[b]
▪ >annually	
o MRD- patient	13%
o MRD+ patient	4%
▪ I am not sure	
o MRD- patient	4%
o MRD+ patient	9%
**A patient is in CR, has undergone ASCT, was initiated on maintenance treatment two years ago and has been continuously MRD- for the duration of maintenance. What is the minimum frequency at which you would initially recommend testing for MRD in bone marrow?**	
▪ ≤6 monthly	-
▪ >6 monthly to annually	45%[c]
▪ >annually	49%[c]
▪ I am not sure	6%
**A patient is in CR, has been MRD- for at least two years, and their treatment has recently (within the past year) been discontinued. What is the minimum frequency at which you would you initially recommend testing MRD in bone marrow?**	
▪ ≤6 monthly	23%
▪ >6 monthly to annually	51%

▪ >annually	17%
▪ I am not sure	9%
**Would you recommend testing for MRD in bone marrow periodically in a patient receiving continuous treatment who is in CR, to check if the patient has attained MRD negativity or to monitor depth of response over time?**	
▪ Yes, in most instances	72%[d]
▪ Yes, under certain conditions; please specify	19%[d,e]
▪ No; it is rarely useful in such a patient	8%
▪ Maybe/I am not sure	2%
**You answered "Yes" in the above question; please indicate the minimum frequency at which you would initially recommend that MRD should be tested in this scenario?**	
▪ ≤6 monthly	8%
▪ >6 monthly to annually	**75%**
▪ >annually	15%
▪ I am not sure	2%

[a]Clustered consensus to assess MRD within six months: 83% (sum of 57% and 26%).

[b]Clustered consensus to assess MRD in MRD+ patients within one year: 87% (sum of 64% and 23%).

[c]Clustered consensus to assess MRD after six months: 94% (sum of 45% and 49%).

[d]Clustered consensus to recommend MRD testing: 91% (sum of 72% and 19%).

[e]Key conditions to assess MRD provided by the respondents: 1) If the patient is eligible for ASCT; 2) If the patient has high-risk disease; 3) Decision to escalate, change, or stop treatment (including toxicity reasons); 4) If this is a clinical trial; 5) If MRD evaluation includes PET-CT.

Percentage in bold denotes consensus was reached.

ASCT, autologous stem cell transplant; CR, complete response; HDT, high-dose therapy; MRD, measurable residual disease

Supplementary Table S6. Treatment discontinuation (Survey 2)

	Respondents (N=53)
**A patient is receiving continuous treatment, has been in CR for at least two years and has expressed desire to discontinue treatment due to adverse drug reactions. The patient has been MRD- in bone marrow and PET/CT- for the past two years. Would you support the patient's decision to cease therapy?**	
▪ Yes; I would support the patient's decision to cease therapy	70%
▪ No; I would not support therapy discontinuation in the above scenario. If you selected this response, briefly describe the scenario(s), if any, in which you would support discontinuation[a]	21%[a]
▪ Maybe/I am not sure	9%
**What is the minimum length of time a patient in CR would need to be continuously MRD- (in bone marrow and on PET/CT) for you to support their decision to discontinue treatment?**	
▪ ≤1 year as demonstrated on at least two occasions. Please indicate your recommended minimum interval between observations	8%[b]
▪ >1–2 years as demonstrated on at least two occasions. Please indicate your recommended minimum interval between observations	17%[c]
▪ >2 years as demonstrated on at least two occasions. Please indicate your recommended minimum interval between observations	51%[d]
▪ I would not support a decision to discontinue treatment regardless. Please provide your reasoning	25%[e]
**In a patient who has ceased therapy and is willing/motivated to have bone marrow tests, how important is long-term MRD testing?**	**Mean rating = 1.8**
▪ 1 (Very important)	53%[f]
▪ 2	34%[f]
▪ 3	6%
▪ 4	-
▪ 5 (Not important)	8%

[a]Key conditions to support treatment discontinuation provided by the respondents: 1) If patient is experiencing treatment intolerance and does not have high risk disease; 2) If no high-risk disease and patient agreed for MRD monitoring; 3) It depends on the personal specific balance of risk-toxicity/benefit; 4) If when new data become available, for example, from the PERSEUS trial showing that discontinuing maintenance in patients who are sustained MRD negative is safe and does not result in progression; 5) If patient has standard risk disease; 6) There is no scientific evidence allowing us to stop therapy, however, I will respect the patient's choice after adequate discussion/information.

[b]Key minimum duration of MRD-negativity provided by the respondents: 1) 3–6 months; 2) 6 months; 3) 12 months.

[c]Key minimum duration of MRD-negativity provided by the respondents: 1) 1 year; 2) If in continuous CR, stop maintenance after 2 years.

[d]Key minimum duration of MRD-negativity provided by the respondents: 1) The longer MRD negative, the better, with a minimum of 2 years; 2) 6 months in the first year of maintenance, annually thereafter.

[e]Key reasons for not supporting treatment discontinuation provided by the respondents: 1) There is insufficient data and no guidelines to support treatment discontinuation based on MRD status; 2) Patients benefit from treatment irrespective of MRD status.

[f]Clustered consensus that long-term MRD testing is important: 87% (sum of 53% and 34%).

ASCT, autologous stem cell transplant; CR, complete response; HDT, high-dose therapy; MRD, measurable residual disease; PET/CT, positron-emission tomography / computed tomography

Supplementary Table S7. Treatment re-initiation following discontinuation (Survey 2)

	Respondents (N=53)
**If relapse was detected in a previously MRD- patient in whom treatment was discontinued, under ideal circumstances, please indicate the initial stage of relapse that you would prefer to re-initiate treatment**	
▪ Biochemical relapse	
○ Standard-risk patient	70%
○ High-risk patient	32%
▪ Clinical relapse	
○ Standard-risk patient	15%
○ High-risk patient	8%
▪ MRD+ relapse	
○ Standard-risk patient	15%
○ High-risk patient	60%

MRD, measurable residual disease

Supplementary Figure S1. Expert panel selection

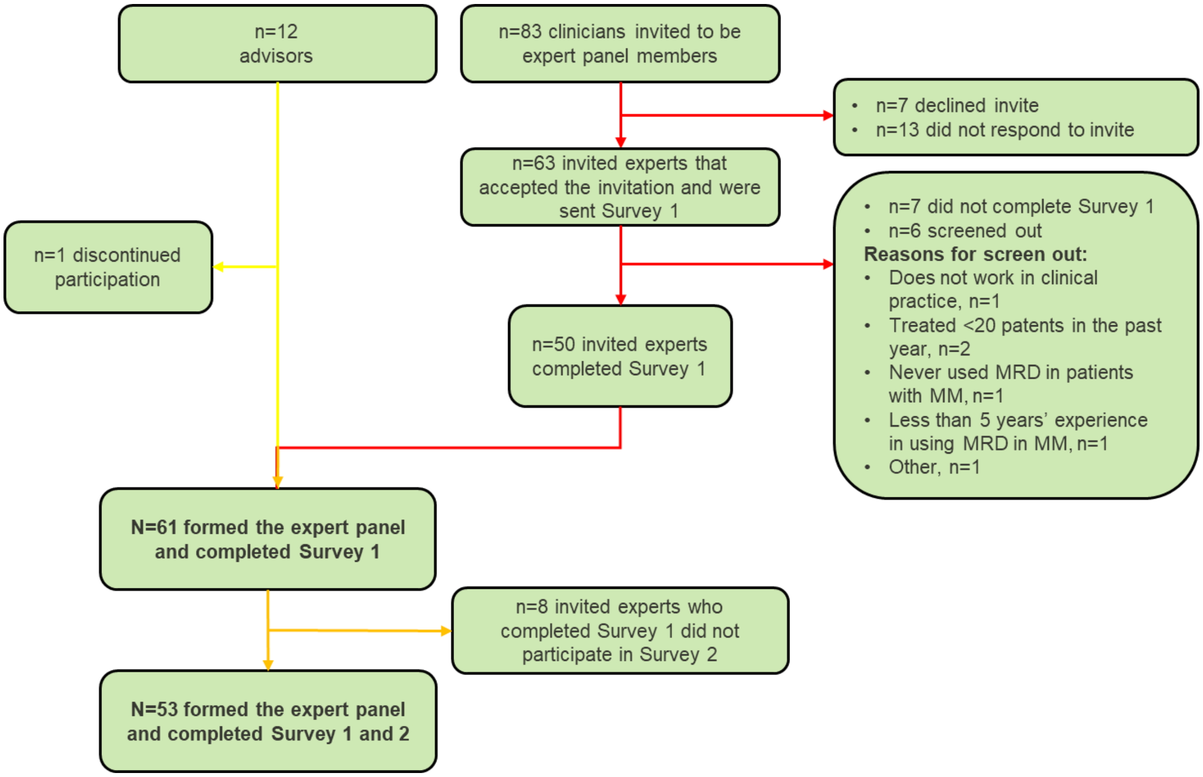

MM, multiple myeloma; MRD, measurable residual disease

Appendix 1: Survey 1 questions

MRD in MM Modified Delphi Initiative: Survey 1

Background information to this modified Delphi initiative

Thank you for participating in this Delphi initiative regarding the use of **measurable residual disease (MRD)** in bone marrow samples (not including positron emission tomography/computed tomography and other forms of functional imaging), in multiple myeloma (MM).

You were personally selected by our advisors to be on a panel of haematological oncologists with significant expertise in the management of patients with MM and the use of MRD.

The goal of this initiative is to use a modified Delphi methodology to help understand where there is consensus or disagreement regarding the use of MRD in MM, particularly concerning assessing response to therapy. This Delphi process will involve up to three rounds of surveys, lasting approximately 30 minutes each. We hope the outputs of this Delphi initiative will provide further guidance to physicians in the management of patients with MM. Ashfield Healthcare Communications will share anonymized and aggregated responses with Adaptive Biotechnologies Corp. and the Delphi advisory panel. The data and findings will be shared with the medical community through, but not limited to, publications including congress abstracts and manuscripts.

All your responses will remain anonymous, except to the third party (Ashfield Healthcare Communications) collating the responses.

Sponsorship:

This Delphi initiative has been supported by Adaptive Biotechnologies Corp. Adaptive Biotechnologies Corp. have provided funding for medical writing support in conducting this survey, compiling the report, and facilitating associated project management and editorial processes. The survey was discussed and developed by the advisors in a face-to-face meeting, which was sponsored by Adaptive Biotechnologies Corp.

Data Privacy Notice

Your *contact details* were obtained through your previous or ongoing working relationships either with a member the Advisory Board or Adaptive Biotechnologies Corp. or were obtained through publicly available sources for the purpose of conducting this survey. The legal basis for the collection and processing of your personal data by Adaptive Biotechnologies Corp. is the General Data Protection Regulation (GDPR) art. 6(1)(f) (legitimate interests) to understand the management of disease in areas of business interest. If you choose to participate in this initiative, your contact details will be

handled in accordance with the GDPR regulations for the purposes of communicating with you regarding this initiative, and may be processed in the European Economic Area or the United States by Adaptive Biotechnologies Corp. or its service providers for this purpose. Your contact details will be retained for as long as necessary to fulfil the purposes above or deleted if you choose not to participate in the survey (unless already held by Adaptive Biotechnologies Corp. in association with other past or ongoing collaborations, as notified to you). Adaptive Biotechnologies Corp. data privacy policy, including contact details for their Data Protection Officer and your legal rights under data privacy law, are available at https://www.adaptivebiotech.com/online-privacy-policy/.

Screening Questions (SQ)

[SQ1] Are you willing to participate in this Delphi initiative? If you qualify, it will involve answering up to three rounds of survey questions (this is Round 1), with each round lasting approximately 30 min.

Select one

[r1] Yes

[r2] No

End of Survey – Participant screens out

Condition: **(SQ1.r2)**

[SQ2] Which of the following best represents the main centre in which you conduct clinical practice?

Select one

[r1] Academic centre/university or teaching hospital

[r2] Government institution

[r3] Large private national institution

[r4] Community hospital (small local hospitals that concentrate on providing a range of services to their local community)

[r5] Private practice

[r99] Other (please provide details)

[r98] I do not work in clinical practice

End of Survey – Participant screens out

Condition: **(SQ2.r98)**

[SQ3] For how many years have you been involved in caring for patients with MM in your capacity as a haematological oncologist?

Select one

[r1] 0 years

[r2] <5 years

[r3] ≥5 – <10 years

[r4] ≥10 – <15 years

[r5] ≥15 – <20 years

[r6] ≥20 – <25 years

[r7] ≥25 – <30 years

[r8] ≥30 – <35 years

[r9] ≥35 – <40 years

[r10] ≥40 – <45 years

[r11] ≥45 – <50 years

[r12] ≥50 years

End of Survey – Participant screens out

Condition: **(SQ3.r1 or SQ3.r2)**

[SQ4] Within the past 12 months, approximately how many patients with MM have you been involved in treating?

Select one

[r1] 0 patients

[r2] ≥1 – <10 patients

[r3] ≥10 – <20 patients

[r4] ≥20 – <30 patients

[r5] ≥30 – <40 patients

[r6] ≥40 – <50 patients

[r7] ≥50 patients

End of Survey – Participant screens out

Condition: **(SQ4.r1 or SQ4.r2 or SQ4.r3)**

[SQ5] Which of the following best describes your experience regarding the use of MRD in MM?

Select one

[r1] I use it in clinical trials, when required by the study protocol

[r2] I use it in my own clinical practice

[r3] I use it both in clinical trials and in my own clinical practice

[r4] I never use MRD in patients with MM

End of Survey – Participant screens out

Condition: **(SQ5.r4)**

Thank you for this information. Please proceed below to the remainder of the survey.

Main survey

The questions are grouped into five categories:

- Perceptions regarding the potential use of MRD in clinical practice in MM
- Use of MRD in MM
- Barriers to routine MRD assessment in MM
- Guidelines concerning the use of MRD in MM.
- Educational needs concerning the use of MRD in MM

Please complete the questionnaire in one session. There are 44 questions, including five case studies; the questionnaire should take approximately 30 minutes to complete. For each question, please select the most appropriate option that you would recommend, insert your answer as free text (**please keep your answers brief**) or assign descriptors, as directed.

Perceptions regarding the potential use of MRD in clinical practice in MM

[Q1] Should MRD testing become part of routine clinical practice in MM?

Select one

[r1] Yes

[r2] No

[r98] Maybe/I don’t know

Click here to see abbreviation definitions

[Q2] When testing for MRD in patients with MM, of the following, what would you recommend as the minimum limit of detection?

Select one

[r1] 10^{-4}

[r2] 10^{-5}

[r3] 10^{-6}

[r98] Other/I don’t know

Click here to see abbreviation definitions

[Q3] In general, how frequently would you recommend testing for MRD in patients with MM in routine clinical practice?

Select one

[r1] One-off testing, as needed

[r2] Every three months

[r3] Every six months

[r4] Every year

[r5] There is no specific time point or frequency at which I use MRD; it depends on patient response over time

[r6] Other (please provide details)

Click here to see abbreviation definitions

[Q4] What type of samples do you normally use to test MRD in your patients with MM, in your routine clinical practice?

Select one

[r1] Peripheral blood

[r2] Bone marrow samples

[r3] Both

Click here to see abbreviation definitions

Use of MRD in MM

Please answer the questions under the following assumptions

- *You have unrestricted access to testing facilities.*
- *There are no barriers limiting your use of MRD.*
- *MRD is assessed in bone marrow samples using either flow cytometry- or PCR/NGS-based methodologies with limits of detection/sensitivities of at least 10^{-5}.*
- *MRD- indicates <10^{-5}; MRD+ indicates 10^{-5}–10^{-3}).*

Five case studies have been developed to provide context when answering the following questions.

Click here to see abbreviation definitions

CASE 1: 61-year-old female, PS 0, R-ISS II. Following four cycles of VRd, the patient achieves a CR (defined as IF- and PC in BM <5%; not stringent CR). She is PET/CT+ at some sites. She is transplant-eligible, and you would like her to undergo HDT-ASCT.

[Q5] Would you recommend testing for MRD prior to HDT-ASCT?

Please provide a brief rationale for your answer.

[r1] Yes

[r2] No

[r3] Maybe/I don’t know

Click here to see abbreviation definitions

Click here to see assumptions

[Q6] You test and find that the patient is MRD+. What would you recommend? (If you selected 'No' to testing MRD in the previous question, please approach this question as if a colleague was seeking your advice concerning this situation).

Select one

[r1] Treating with additional cycles of VRd until she is MRD-, before undergoing ASCT

[r2] Continuing directly to ASCT; MRD results would not impact my decision on moving directly to ASCT

[r98] I don't know

Click here to see abbreviation definitions

Click here to see Case 1

Click here to see assumptions

Your patient undergoes HDT-ASCT. You confirm that she is in 'conventional' CR (defined as IF- and PC in BM <5%), and you would like to initiate maintenance therapy.

[Q7] Would you recommend re-assessing your patient's PET/CT status?

Select one

[r1] Yes

[r2] No

[r98] Maybe/I don't know

Click here to see abbreviation definitions

Click here to see Case 1

Click here to see assumptions

[Q8] Would you recommend confirming your patient's MRD status?

Select one

[r1] Yes

[r2] No

[r98] Maybe/I don't know

Click here to see abbreviation definitions

Click here to see Case 1

Click here to see assumptions

[Q9] You decide to confirm MRD status. How long would you recommend waiting post-ASCT before taking a fresh bone marrow sample for MRD assessment? (If you selected 'No' to testing MRD in the previous question, please approach this question as if a colleague was seeking your advice concerning this situation).

Select one

[r1] Less than three months

[r2] Three months

[r3] Four months

[r4] Five months

[r5] Six months

[r6] Twelve months

[r99] Other (please provide details)

Click here to see abbreviation definitions

Click here to see Case 1

Click here to see assumptions

[Q10] Is the information you learn from PET/CT and MRD complementary?

Select one

[r1] Both provide important complementary data; I would use them together

[r2] Both provide important data but I only need one or the other, not both

[r3] I would do PET/CT first and then, only if it is negative, would I test MRD

[r4] I would test MRD first and then, only if it is negative, would I conduct PET/CT

[r98] Maybe/I don't know

Click here to see abbreviation definitions

Click here to see Case 1

Click here to see assumptions

CASE 1 CONTINUED: Your patient is initiated on maintenance therapy with lenalidomide post-ASCT.

[Q11] Your patient remains in CR. You decide to assess MRD periodically. How frequently would you recommend assessing MRD during maintenance?

Select one

[r1] Every three months

[r2] Every six months

[r3] Every year

[r4] I would not assess MRD regularly

[r99] Other (please provide details)

Click here to see abbreviation definitions

Click here to see Case 1

Click here to see assumptions

CASE 1 CONTINUED: Your patient has been on maintenance therapy for two years. She remains in conventional CR defined as IF- and PC in BM <5% (details of additional test results are given in the questions below). She is interested in discontinuing therapy as she has been experiencing some mild gastrointestinal-related side effects and is not keen to start alternative treatments. She wants to know if you would support her proposal to discontinue therapy.

[Q12] You would like to support her wish to discontinue therapy. Please select all the scenarios in which you would be content, per your experience as an MM expert, to support therapy discontinuation.

Select all that apply

[r1] Scenario 1: Patient has been in CR for the past two years (no other tests are available)

[r2] Scenario 2: Patient is in CR with PET/CT- results for the past two years (patient has not been tested for MRD)

[r3] Scenario 3: Patient is in CR with MRD- results for the past two years (patient has not been tested for PET/CT status)

[r4] Scenario 4: Patient is in CR with MRD- and PET/CT- results for the past two years

[r5] Scenario 5: I would not support therapy discontinuation in any of the above scenarios. If you selected this response, briefly describe the scenario(s), if any, in which you would support discontinuation:

Click here to see abbreviation definitions

Click here to see Case 1

Click here to see assumptions

[Q13] Question 13 of 44 If you would use MRD status in guiding whether you would support therapy discontinuation, what is the minimum amount of time that she would need to be continuously MRD- before you would support therapy discontinuation?

Select one

[r1] One test indicating MRD negativity (at a single time point) is sufficient

[r2] A minimum of two tests demonstrating three months of MRD- is sufficient

[r3] A minimum of two tests demonstrating six months of MRD- is sufficient

[r4] A minimum of two tests demonstrating one year of MRD- is sufficient

[r5] A minimum of two tests demonstrating two years of MRD- is sufficient

[r99] Other (please provide details)

[r98] I would never use MRD results when making the decision to discontinue therapy

[r97] I would never support therapy discontinuation in any circumstance

Click here to see abbreviation definitions

Click here to see Case 1

Click here to see assumptions

Case 1 continued: The patient discontinues therapy.

[Q14] At what frequency would you recommend that your patient be tested for MRD?

Select one

[r1] Every three months

[r2] Every six months

[r3] Every year

[r4] I would not assess MRD regularly

[r5] I would not assess MRD in this patient

[r99] Other (please provide details)

Click here to see abbreviation definitions

Click here to see Case 1

Click here to see assumptions

[Q15] Provided she remains MRD-, for how long would you recommend continuing testing her for MRD?

Select one

[r1] For a finite period of time (please provide the amount of time)

[r2] For as long as she is off treatment

[r3] I would not assess MRD regularly

Click here to see abbreviation definitions

Click here to see Case 1

Click here to see assumptions

[Q16] Please rank (1 = most important, 4 = least important) the following test results by their relative importance in indicating that your patient remains disease-free.

Click or drag each item into a rank position.

[r1] IF-

[r2] PET/CT-

[r3] MRD-

[r4] PET/CT- / MRD-

Click here to see abbreviation definitions

Click here to see Case 1

Click here to see assumptions

CASE 1 CONTINUED: At the 2-year assessment, the patient is MRD+.

[Q17] At what point would you recommend to re-initiate therapy?

Select one

[r1] Immediately

[r2] It would depend on the MRD level (please provide the MRD threshold (10^{-4}, 10^{-5}, or 10^{-6}) at which you would re-initiate therapy:)

[r3] I would continue to monitor the patient for sustained ‘conventional’ CR and not re-initiate therapy until the patient has a clinical relapse

[r99] Other (please provide details)

Click here to see abbreviation definitions

Click here to see Case 1

Click here to see assumptions

[Q18] You determine that your patient’s risk status has progressed to high-risk. Would you use MRD to assist you in making therapeutic decisions in this patient?

Select one

[r1] Yes

[r2] No

[r98] Maybe/I don’t know

Click here to see abbreviation definitions

Click here to see Case 1

Click here to see assumptions

CASE 2: 75-year-old male, newly diagnosed, PS 1. Following eight cycles of VRd induction therapy, the patient achieved ‘conventional’ CR (defined as IF- and PC in BM <5%). The patient is transplant-ineligible and currently on a lenalidomide maintenance therapy, which he is tolerating well.

[Q19] Would you recommend testing your patient for MRD?

Please provide a brief rationale for your answer

[r1] Yes

[r2] No

[r3] Maybe/I don’t know

Click here to see abbreviation definitions

Click here to see assumptions

[Q20] Would you consider ceasing treatment?

Please select the extent to which you agree with each statement using the 1–5 Likert scale of strongly agree to strongly disagree.

[r1] Yes; the patient is in CR and this is sufficient

[r2] No; the patient is tolerating therapy

[r3] I would use MRD as part of the consideration for ceasing treatment

Click here to see abbreviation definitions

Click here to see Case 2

Click here to see assumptions

[Q21] You test for MRD, and your patient is MRD+ despite being in CR. What would you recommend? (If you selected 'No' to testing MRD in the earlier question, please approach this question as if a colleague was seeking your advice concerning this situation).

Select one

[r1] Ceasing treatment; CR is sufficient

[r2] Continuing the current maintenance regimen

[r3] Modifying the current regimen or switching therapies to get the patient to MRD- status

[r99] Other (please provide details)

Click here to see abbreviation definitions

Click here to see Case 2

Click here to see assumptions

[Q22] You test for MRD, and your patient is MRD-. What would you recommend? (If you selected 'No' to testing MRD in the earlier question, please approach this question as if a colleague was seeking your advice concerning this situation).

Select one

[r1] Ceasing treatment

[r2] Continuing the current maintenance regimen

[r3] Performing a re-evaluation after x months; please state how many months you would recommend continuing the regimen prior to re-evaluation

[r4] Other (please provide details)

Click here to see abbreviation definitions

Click here to see Case 2

Click here to see assumptions

CASE 3: 65-year-old male, PS 2, who is transplant-eligible. The patient is receiving maintenance therapy with lenalidomide and has achieved a VGPR as determined by paraprotein level.

[Q23] Would you recommend testing your patient for MRD?

Please provide a brief rationale for your answer.

[r1] Yes

[r2] No

[r98] Maybe/I don't know

Click here to see abbreviation definitions

Click here to see assumptions

[Q24] You decide to test your patient's MRD status. How frequently would you recommend that your patient be evaluated for MRD? (If you selected 'No' to testing MRD in the previous question, please approach this question as if a colleague was seeking your advice concerning this situation).

Select one

[r1] Every three months

[r2] Every six months

[r3] Every twelve months

[r4] There is no specific time point or frequency at which I use MRD; it depends on patient response

[r99] Other (please provide details)

Click here to see abbreviation definitions

Click here to see Case 3

Click here to see assumptions

[Q25] You test for MRD, and your patient is MRD-, despite only achieving a VGPR. What would you recommend? (If you selected ‘No’ to testing MRD in the earlier question, please approach this question as if a colleague was seeking your advice concerning this situation).

Select one

[r1] Retesting your patient’s MRD status at the next scheduled MRD-testing appointment

[r2] Retesting the patient’s paraprotein level to see if it has reduced or is negative

[r99] Other (please provide details)

[r98] I don’t know

Click here to see abbreviation definitions

Click here to see Case 3

Click here to see assumptions

CASE 4: 65-year-old male, PS 1, who has previously undergone ASCT. The patient recently relapsed and has since been started on and is currently receiving continuous treatment with Dara-Vd to ‘conventional’ CR (defined as IF- and PC in BM <5%). He has had one line of therapy previously and was not refractory.

[Q26] Would you recommend testing your patient for MRD?

Please provide a brief rationale for your answer.

[r1] Yes

[r2] No

[r3] Maybe/I don’t know

Click here to see abbreviation definitions

Click here to see assumptions

[Q27] Would you consider ceasing treatment?

Please select the extent to which you agree with each statement using the 1–5 Likert scale of strongly agree to strongly disagree.

[r1] Yes; the patient is in CR and this is sufficient

[r2] Yes; the patient is in CR, he is experiencing side effects and wants to stop therapy

[r33] No; the patient is tolerating therapy

[r3] I would use MRD as part of the consideration for ceasing treatment

Click here to see abbreviation definitions

Click here to see Case 4

Click here to see assumptions

[Q28] You test for MRD, and your patient is MRD+, despite being in CR. What would you recommend? (If you selected 'No' to testing MRD in the earlier question, please approach this question as if a colleague was seeking your advice concerning this situation).

Select one

[r1] Ceasing treatment

[r2] Continuing the current maintenance regimen

[r3] Performing a re-evaluation after x months; please state how many months you would recommend continuing the regimen prior to re-evaluation

[r99] Other (please provide details)

Click here to see abbreviation definitions

Click here to see Case 4

Click here to see assumptions

[Q29] You test for MRD, and your patient is MRD-. What would you recommend? (If you selected 'No' to testing MRD in the earlier question, please approach this question as if a colleague was seeking your advice concerning this situation).

Select one

[r1] Ceasing treatment

[r2] Continuing treatment

[r99] Other (please provide details)

Click here to see abbreviation definitions

Click here to see Case 4

Click here to see assumptions

CASE 5: Your patient has previously undergone ASCT and is now undergoing maintenance therapy with lenalidomide, and is currently in CR; they want to cease maintenance therapy. Please indicate whether the following patient characteristics would impact your interest in knowing your patient's MRD status prior to the patient ceasing treatment. Please note details of the patient (e.g. age, risk, performance status) have not been provided, as the following questions are designed to explore how your clinical recommendations vary according to different clinical settings

---

[Q30] Would patient/disease category impact your interest in knowing your patient's MRD status prior to supporting their request to cease maintenance therapy?

Select all that apply

[r1] Yes; I would want to know their MRD status if the patient was **newly diagnosed**.

[r2] Yes; I would want to know their MRD status if the patient had **relapsed.**

[r3] Yes; I would want to know their MRD status if the patient was **transplant-eligible**.

[r4] Yes; I would want to know their MRD status if the patient was **transplant-ineligible.**

[r5] No; patient/disease category would not impact my interest in knowing their MRD status because I would want to know their MRD status regardless

[r6] No; patient/disease category would not impact my interest in knowing their MRD status because MRD status would not impact my treatment recommendations

Click here to see abbreviation definitions

Click here to see assumptions

[Q31] Would patient ECOG performance status or patient fitness/frailty impact your interest in knowing your patient's MRD status prior to supporting their request to cease maintenance therapy?

Select one

[r1] Yes; I would want to know their MRD status if the patient was characterised as certain ECOG statuses or whether they were fit or frail

[r3] No; ECOG status and patient fitness/frailty would not impact my interest in knowing their MRD status because I would want to know their MRD status regardless

[r4] No; ECOG status and patient fitness/frailty would not impact my interest in knowing their MRD status because MRD status would not impact my treatment recommendations

Click here to see abbreviation definitions

Click here to see Case 5

Click here to see assumptions

[Q31a] You selected "Yes; I would want to know their MRD status if the patient was characterised as certain ECOG statuses or whether they were fit or frail".

Condition: **(Q31.r1)**

Please indicate which categories apply as relevant.

[r1] PS 0

[r2] PS 1

[r3] PS 2

[r4] PS 3

[r5] Fit

[r6] Elderly / Frail

Click here to see abbreviation definitions

Click here to see Case 5

[Q32] Would patient age impact your interest in knowing their MRD status prior to supporting the patient in their request to cease maintenance therapy?

Select one

[r1] Yes; I would want to know their MRD status if the patient was within certain age brackets

[r2] No; patient age would not impact my interest in knowing their MRD status because I would want to know their MRD status regardless

[r3] No; patient age would not impact my interest in knowing their MRD status because MRD status would not impact my treatment recommendations

Click here to see abbreviation definitions

Click here to see Case 5

Click here to see assumptions

[Q32a] You selected "Yes; I would want to know their MRD status if the patient was within certain age brackets".

Condition: **(Q32.r1)**

Please indicate which categories apply as relevant.

[r1] <40 years

Condition: **(Q32.r1)**

[r2] 40–49 years

[r3] 50–59 years

[r4] 60–69 years

[r5] 70–79 years

[r6] 80–89 years

[r7] ≥90 years

Click here to see abbreviation definitions

Click here to see Case 5

Click here to see assumptions

[Q33] Would patient R-ISS risk category impact your interest in knowing their MRD status prior to supporting the patient in their request to cease maintenance

therapy?

Select all that apply.

[r1] Yes; I would want to know their MRD status if the patient was within the following risk category — **Standard**

[r2] Yes; I would want to know their MRD status if the patient was within the following risk category — **High**

[r3] Yes; I would want to know their MRD status if the patient was within the following risk category — **High with R-ISS III**

[r4] No; patient risk category would not impact my interest in knowing MRD because I would want to know their MRD status regardless

[r5] No; patient risk category would not impact my interest in knowing their MRD status because MRD status would not impact my treatment recommendations

Click here to see abbreviation definitions

Click here to see Case 5

Click here to see assumptions

[Q34] Question 34 of 44 Would patient treatment line/relapse history impact your interest in knowing their MRD status prior to supporting the patient in their request to cease maintenance therapy?

Select all that apply

[r1] Yes; I would want to know their MRD status if the patient was within the following category, in terms of treatment line/relapse history — **First line**

[r2] Yes; I would want to know their MRD status if the patient was within the following category, in terms of treatment line/relapse history — **Following first relapse**

[r3] Yes; I would want to know their MRD status if the patient was within the following category, in terms of treatment line/relapse history — **Following second relapse**

[r4] Yes; I would want to know their MRD status if the patient was within the following category, in terms of treatment line/relapse history — **Following third+ relapse**

[r5] No; patient treatment line/relapse history would not impact my interest in knowing their MRD status because I would want to know their MRD status regardless

[r6] No; patient treatment line/relapse history would not impact my interest in knowing their MRD status because MRD status would not impact my treatment recommendations

Click here to see abbreviation definitions

Click here to see Case 5

Click here to see assumptions

Thank you for answering the case study-related questions. The last few questions focus on exploring your current opinions on the following topics “Barriers to Routine MRD Assessment in MM”, “Use of Guidelines Concerning the Use of MRD in MM”, and “Educational Needs Regarding the Use of MRD in MM”.

Barriers to Routine MRD Assessment in MM

[Q35] Please rate the impact of the following barriers in terms of hindering you in routine practice regarding use of MRD in MM.

Please select from the following descriptors: very high impact, high impact, moderate impact, low impact, or no impact.

Row:

[r1] Reimbursement

[r2] Access to testing facility

[r3] Difficulties in obtaining high-quality bone marrow samples

[r4] Lack of strong supporting scientific evidence

[r5] Lack of guidelines

[r6] Need for testing at multiple time points

[r7] Lack of patient consent to periodic bone marrow sampling

[r8] Lack of baseline sample hindering MRD testing

Click here to see abbreviation definitions

[Q36] Please rate the impact of the following barriers in terms of hindering clinicians who treat MM in your country regarding use of MRD in MM.

Please use the following descriptors: very high impact, high impact, moderate impact, low impact, or no impact.

Row:

[r1] Reimbursement

[r2] Access to testing facility

[r3] Difficulties in obtaining high-quality bone marrow samples

[r4] Lack of strong supporting scientific evidence

[r5] Lack of guidelines

[r6] Need for testing at multiple time points

[r7] Lack of patient consent to periodic bone marrow sampling

[r8] Lack of baseline sample hindering MRD testing

Click here to see abbreviation definitions

Use of Guidelines Concerning the Use of MRD in MM

[Q37] Please provide the name(s) of the prognostic and treatment guidelines that you use:

Be specific, if possible.

Row:

[r1] Prognostic:

[r2] Treatment:

Click here to see abbreviation definitions

[Q38] Is MRD covered in the prognostic or treatment guidelines that you use?

Select one

Row:

[r1] MRD is covered in the prognostic guidelines that I use

[r2] MRD is covered in the treatment guidelines that I use

[r3] MRD is covered in both the prognostic and treatment guidelines I use

[r4] MRD is not covered in the prognostic or treatment guidelines I use

[r5] I do not use guidelines

[r6] I’m not sure

Click here to see abbreviation definitions

[Q39] If you use guidelines other than prognostic or treatment guidelines describing the use of MRD in MM, please specify the guideline type below.

Click here to see abbreviation definitions

[Q40] Educational Needs Regarding the Use of MRD in MM Question 40 of 44 Do you feel that you are suitably informed regarding the use of MRD in MM such that you can use it with confidence in a variety of clinical situations?

Select one

Row:

[r1] Yes

[r2] No

Click here to see abbreviation definitions

[Q41] In which of the following would you like to receive more education/resources?

Please select all that apply.

Row:

[r1] Methodologies available for testing MRD

[r2] Use of MRD in prognostication

[r3] Use of MRD in informing treatment decisions

[r4] Guidelines available on the clinical use of MRD in MM

[r5] Other (please provide details)

Click here to see abbreviation definitions

[Q42] Do you feel that clinicians in your country are suitably informed regarding the use of MRD in MM such that they can use it with confidence in a variety of clinical situations?

Select one

Row:

[r1] Yes

[r2] No

Click here to see abbreviation definitions

[Q43] In which of the following categories do you feel that clinicians in your country would benefit from more education/resources regarding MRD in MM? Please select all that apply.

Select all that apply

Row:

[r1] Methodologies available for testing MRD

[r2] Use of MRD in prognostication

[r3] Use of MRD in informing treatment decisions

[r4] Guidelines available on the clinical use of MRD in MM

[r99] Other (please provide details)

Click here to see abbreviation definitions

[Q44] Would you like to be acknowledged in any outputs from this Delphi initiative? (Note, irrespective of your response, your specific feedback will remain anonymous, except to the third party (Ashfield Healthcare Communications) collating the responses. If you select "Yes", your name would be included in the acknowledgement section of the potential publications to indicate that you participated in the Expert Panel).

Row:

[r1] Yes

[r2] No

Click here to see abbreviation definitions

Many thanks for your participation in this questionnaire. Your responses to this first-round Delphi survey will be used to develop the second survey, which will be sent to you within the coming weeks.

Appendix 2: Survey 2 questions

MRD in MM Modified Delphi Initiative: Survey 2

Background information to this modified Delphi initiative

Thank you for completing Survey 1, the results of which have been emailed to you previously. For your convenience, the results of Survey 1 are also available via the following link: *Link removed*. We now welcome you to complete Survey 2, which was developed based on the findings of Survey 1.

As a reminder, the goal of this initiative is to use a modified Delphi methodology to help understand where and why there is consensus or no consensus regarding the use of measurable residual disease (MRD) in multiple myeloma (MM), particularly with respect to assessing response to therapy. This modified Delphi process will involve up to three surveys, lasting approximately 30 minutes each; you have already completed the first survey. We hope the outputs of this modified Delphi initiative will provide further guidance to physicians in the management of patients with MM. Ashfield Health will share anonymised and aggregated responses with Adaptive Biotechnologies Corp. and the Delphi advisory panel. The data and findings will be shared with the medical community through publications, including congress, abstracts and manuscripts.

As was the case in Survey 1, all your responses will remain anonymous, except to the third party (Ashfield Health) collating the responses.

Sponsorship:

This Delphi initiative has been supported by Adaptive Biotechnologies Corp. Adaptive Biotechnologies Corp. has provided funding for medical writing support in conducting this survey, compiling the report, and facilitating associated project management and editorial processes. The content of Survey 2 was discussed and developed by the advisors in a virtual meeting that was sponsored by Adaptive Biotechnologies Corp., and was based on the results of Survey 1.

Data Privacy Notice

Your contact details were initially obtained through your previous or ongoing working relationships either with a member of the Advisory Board or Adaptive Biotechnologies Corp. or were obtained through publicly available sources for the purpose of conducting this survey. The legal basis for the collection and processing of your personal data by Adaptive Biotechnologies Corp. is the General Data Protection Regulation (GDPR) art. 6(1)(f) (legitimate interests) to understand the management of disease in areas of business interest. If you choose to continue participating in this initiative, your contact details will be handled in accordance with the GDPR regulations for the purposes of communicating with you regarding this initiative, and may be processed in the European Economic Area or the United States by Adaptive Biotechnologies Corp. or its service providers for this purpose. Your contact details will be retained for as long as necessary

to fulfil the purposes above or deleted if you choose not to participate in the survey (unless already held by Adaptive Biotechnologies Corp. in association with other past or ongoing collaborations, as notified to you). Adaptive Biotechnologies Corp. data privacy policy, including contact details for their Data Protection Officer and your legal rights under data privacy law, are available at https://www.adaptivebiotech.com/online-privacy-policy/.

Before starting Survey 2, we would appreciate if you could **familiarise yourself** with:

- The results of Survey 1: *Link removed*.
- The following literature articles and abstracts that report results of recent major clinical trials in MM (listed in alphabetical order per trial name):
    - Mateos, MV et al. *Lancet* 2020;395:132–41 **(ALCYONE trial)**
        - Daratumumab plus bortezomib, melphalan and prednisone versus bortezomib, melphalan and prednisone alone in patients with newly diagnosed multiple myeloma who were ineligible for autologous stem-cell transplantation https://doi.org/10.1016/S0140-6736(19)32956-3
    - Moreau, P et al. *Blood* 2019;134:692 (**Cassiopeia study – Cassiopet Companion study)**
        - Evaluation of the prognostic value of ^{18}F-fluorodeoxyglucose (FDG) positron emission tomography/computed tomography (PET/CT) at diagnosis and follow-up in transplant-eligible patients with newly diagnosed multiple myeloma: results of the Cassiopet Companion study https://doi.org/10.1182/blood-2019-123143
    - Palumbo, A et al. *N Engl J Med* 2016;375:754–66 (**CASTOR trial)**
        - Daratumumab plus bortezomib and dexamethasone versus bortezomib and dexamethasone alone in patients with relapsed, or relapsed and refractory multiple myeloma https://www.nejm.org/doi/full/10.1056/nejmoa1606038
    - Mateos, MV et al. *Clin Lymphoma Myeloma Leuk* 2020;20:509–18 (**CASTOR trial)**
        - Three-year follow-up of CASTOR https://doi.org/10.1016/j.clml.2019.09.623
    - Avet-Loiseau, H et al. *J Clin Onc* 2021; ahead of print (**CASTOR and POLLUX)**
        - Evaluation of sustained MRD negativity with daratumumab-combination regimens in patients with relapsed and/or refractory multiple myeloma in the POLLUX and CASTOR trials https://ascopubs.org/doi/full/10.1200/JCO.20.01814
    - Zamagni, E et al. Poster from ASH 2020 virtual congress (**FORTE trial**)
        - Impact of imaging FDG-PET/CT MRD assessment on outcomes and matching with bone marrow techniques in transplant-eligible patients with newly diagnosed multiple myeloma

    https://ash.confex.com/ash/2020/webprogram/Paper137412.html

- Zamagni, E et al. Oral presentation from EHA 2020 virtual congress (**FORTE trial**)
  - MRD evaluation by PET/CT according to Deauville criteria combined with bone marrow techniques in newly diagnosed transplant eligible multiple myeloma patients https://library.ehaweb.org/eha/2020/eha25th/295027/elena.zamagni.mrd.evaluation.by.pet.ct.according.to.deauville.criteria.html?f=listing%3D0%2Abrowseby%3D8%2Asortby%3D1%2Asearch%3DMRD+EVALUATION+BY+PET%2FCT+ACCORDING+TO+DEAUVILLE+CRITERIA+COMBINED+WITH+BONE+MARROW+TECHNIQUES+IN+NEWLY+DIAGNOSED+TRANSPLANT+ELIGIBLE+MULTIPLE+MYELOMA+PATIENTS+ENROLLED+IN+THE+PHASE+II+FORTE+TRIAL
- Voorhees, PM et al. *Blood* 2020;136:936–45 **(GRIFFIN trial)**
  - Daratumumab, lenalidomide, bortezomib and dexamethasone versus lenalidomide, bortezomib, and dexamethasone in transplant-eligible patients with newly diagnosed multiple myeloma https://doi.org/10.1182/blood.2020005288
- Moreau, P et al. *J Clin Onc* 2017;35:2911–18 (**IFM 2009 trial**)
  - Prospective evaluation of magnetic resonance imaging and FDG-PET/CT at diagnosis and before maintenance therapy in symptomatic patients with multiple myeloma included in the IFM/DFCI 2009 trial https://ascopubs.org/doi/10.1200/JCO.2017.72.2975
- Perrot, A et al. *Blood* 2018;132:2456–64 (**IFM 2009 trial**)
  - Induction therapy with three cycles of bortezomib, lenalidomide and dexamethasone (VRD), followed by consolidation therapy with either five additional cycles of VRD or high-dose melphalan plus stem-cell transplantation followed by two additional cycles of VRD in patients with newly diagnosed multiple myeloma: analysis of MRD samples with next-generation sequencing https://doi.org/10.1182/blood-2018-06-858613
- Perrot, A et al. Oral presentation at ASH 2020 virtual congress (**IFM 2009 trial**)
  - Early versus late autologous stem cell transplant in newly diagnosed multiple myeloma: long-term follow-up analysis https://ash.confex.com/ash/2020/webprogram/Paper134538.html
- Facon, T et al. *N Engl J Med* 2019;380:2104–15 **(MAIA trial)**
  - Daratumumab plus lenalidomide and dexamethasone versus lenalidomide and dexamethasone alone in patients with newly diagnosed multiple myeloma who were ineligible for autologous stem-cell transplantation https://www.nejm.org/doi/full/10.1056/NEJMoa1817249

- Dimopoulos, MA et al. *N Engl J Med* 2016;375:1319–31 **(POLLUX trial)**
  - Daratumumab plus lenalidomide and dexamethasone versus lenalidomide and dexamethasone alone in patients with relapsed or refractory multiple myeloma who had received one or more lines of previous therapy

https://www.nejm.org/doi/full/10.1056/NEJMoa1607751

- Bahlis, NJ et al. *Leukemia* 2020;34:1875–84 **(POLLUX trial)**
  - Extended follow-up of POLLUX https://www.nature.com/articles/s41375-020-0711-6

Abbreviations

ASCT, autologous stem cell transplant; BM, bone marrow; CR, complete response; ECOG, Eastern Cooperative Oncology Group; HDT, high-dose therapy; IF, immuno-fixation; IHC, immunohistochemistry; IMWG; International Myeloma Working Group; MRD, measurable residual disease; MM, multiple myeloma; NGS, next generation sequencing; PC, plasma cells; PCR, polymerase chain reaction; PET/CT, positron-emission tomography/computed tomography; R-ISS, Revised International Staging System; VGPR, very good partial response

---

Pre-survey questions (PSQ; no screening element)

[PSQ1] Have you familiarised yourself with the results of Survey 1 and suggested literature materials

Select one

Row:

[r1] Yes

[r2] No

[PSQ2] What was your overall impression of the responses in Survey 1 and did you find any of the responses particularly interesting?

Main survey

Questions in Survey 2 are grouped into six categories:

- Use of MRD testing in different clinical scenarios (spanning patients of different ages, transplant eligibility statuses, medical histories and fitness levels)
- Frequency of MRD testing
- Continuous treatment as the norm
- Treatment discontinuation

- Treatment re-initiation following discontinuation
- Factors that determine interest in testing for MRD

Please complete the questionnaire in one session.

There is a maximum of 34 questions including follow-up questions. For each question, please select the most appropriate option(s) in your opinion, insert your answer as free text or assign or rank descriptors, as indicated.

Please answer all questions under the following assumptions:

- *You have **unrestricted access (which includes full reimbursement)** to testing equipment and facilities.*
- *There are **no barriers (including logistical and technical barriers)** limiting your use of MRD.*
- *Unless specified otherwise, **MRD testing refers to testing in bone marrow** using either flow cytometry- or PCR/NGS-based methodologies with limits of detection/sensitivities of at least 10^{-5}; as such, MRD+ indicates detection of at least one tumour cell in equal to or less than 100,000 healthy cells*
- *For the purposes of this survey, please assume that patients who were MRD+ had MRD values of no greater than one tumour cell in 1000 healthy cells.*

Please proceed below to the survey.

Use of MRD testing in different clinical scenarios

[Q1] A patient is in CR and is being considered for HDT-ASCT. When would you recommend testing MRD in bone marrow?

Select one

[r1] I would only recommend testing for MRD after induction therapy but before HDT-ASCT

[r2] I would recommend testing for MRD after induction therapy, before HDT-ASCT, and if the patient is MRD+ then I would also recommend testing after HDT-ASCT

[r3] I would recommend testing for MRD after induction therapy, before HDT-ASCT; I would also recommend testing for MRD after HDT-ASCT, regardless of the MRD test result after induction therapy

[r4] I would only recommend testing for MRD after HDT-ASCT, before starting maintenance

[r5] I would recommend testing for MRD after HDT-ASCT, before starting maintenance treatment, and if the patient is MRD+ then I would also recommend testing during maintenance

[r6] I would recommend testing for MRD after HDT-ASCT, before starting maintenance treatment; I would also recommend testing for MRD during maintenance regardless of the MRD test result after HDT-ASCT (before starting maintenance)

[r7] I would only recommend testing for MRD after HDT-ASCT, during maintenance

[r8] I would recommend testing for MRD at multiple time points (not listed above) in this scenario; please state your recommended time points

[r9] I would not recommend MRD in this situation

[r99] I am not sure

CR: IF- in serum and urine and PC in BM <5%

Click here to see abbreviation definitions

Click here to see assumptions

Click here to see literature articles and abstracts

Click here to see survey 1 data

[Q2] You answered that you would only recommend testing for MRD in bone marrow before HDT-ASCT in the previous question, please explain your reasoning briefly

Condition: **(Q1.r1)**

Click here to see abbreviation definitions

Click here to see assumptions

Click here to see literature articles and abstracts

Click here to see survey 1 data

[Q3] You answered that you would only recommend testing for MRD in bone marrow after HDT-ASCT in the previous question, please explain your reasoning

briefly

Condition: **(Q1.r4 or Q1.r5 or Q1.r6 or Q1.r7)**

Click here to see abbreviation definitions

Click here to see assumptions

Click here to see literature articles and abstracts

Click here to see survey 1 data

[Q4] You answered that you would initially recommend testing for MRD in bone marrow both before and after HDT-ASCT in the previous question, please explain your reasoning briefly

Condition: **(Q1.r3)**

Click here to see abbreviation definitions

Click here to see assumptions

Click here to see literature articles and abstracts

Click here to see survey 1 data

[Q5] You answered you would NOT recommend testing for MRD in bone marrow in the previous question, please explain your reasoning briefly

Condition: **(Q1.r9)**

Click here to see abbreviation definitions

Click here to see assumptions

Click here to see literature articles and abstracts

Click here to see survey 1 data

[Q6] A patient is in CR and is being considered for HDT-ASCT; the patient was PET/CT+ in some locations at baseline. When would you recommend that the patient be tested using PET/CT?

[r1] I would recommend PET/CT before HDT-ASCT but not after

[r2] I would recommend PET/CT after HDT-ASCT but not before

[r3] I would recommend PET/CT both before and after HDT-ASCT

[r4] I would recommend first testing MRD in bone marrow before HDT-ASCT and if positive, only then would I consider the clinical need and timing of PET/CT

[r5] I would not perform PET/CT at all

[r6] I am not sure

CR: IF- in serum and urine and PC in BM <5%

Click here to see abbreviation definitions

Click here to see assumptions

Click here to see literature articles and abstracts

Click here to see survey 1 data

[Q7] A patient in CR is being considered for HDT-ASCT; the patient was PET/CT+ in some sites at baseline. Given results of the CASIOPET Companion study, prior to HDT-ASCT, which test is more of a clinical priority in your opinion — MRD testing in bone marrow or PET/CT?

[r1] MRD in bone marrow

[r2] PET/CT

[r3] Both are of equal priority; one does not outrank the other

[r4] I am not sure

CR: IF- in serum and urine and PC in BM <5%

Click here to see abbreviation definitions

Click here to see assumptions

Click here to see literature articles and abstracts

Click here to see survey 1 data

[Q8] Please indicate your level of agreement with the following statement:

‘In a patient who is in CR who was previously PET/CT+ at baseline, testing both MRD in bone marrow and PET/CT status prior to HDT-ASCT can provide clinically important information.'

Please consider the results of the CASIOPET Companion study in your answer.

Drag the slider to a point on the scale.

Choice:

[ch1] 1 - Strongly agree

[ch2] 2 - Somewhat agree

[ch3] 3 - Neither agree nor disagree

[ch4] 4 - Somewhat disagree

[ch5] 5 - Strongly disagree

Click here to see abbreviation definitions

Click here to see assumptions

Click here to see literature articles and abstracts

Click here to see survey 1 data

[Q9] Please indicate your level of agreement with the following statement:

'In a patient who is in CR who was previously PET/CT+ at baseline, testing both MRD in bone marrow and PET/CT status after HDT-ASCT can provide clinically important information’

Please consider the results of the CASIOPET Companion study in your answer.

Drag the slider to a point on the scale.

Choice:

[ch1] 1 - Strongly agree

[ch2] 2 - Somewhat agree

[ch3] 3 - Neither agree nor disagree

[ch4] 4 - Somewhat disagree

[ch5] 5 - Strongly disagree

Click here to see abbreviation definitions

Click here to see assumptions

Click here to see literature articles and abstracts

Click here to see survey 1 data

[Q10] In a patient who is in CR who was previously PET/CT+ at baseline, given the results of the CASIOPET Companion study, prior to HDT-ASCT, what is your opinion on the use of MRD testing in bone marrow and PET/CT? Question 6 of 29

[r1] Both provide important complementary data. I would use them together; the order of testing is not relevant

[r2] Both provide important complementary data, but I would test for MRD first and then consider PET/CT

[r3] Both provide important complementary data, but I would do PET/CT first and then consider testing MRD

[r4] Both provide important data, but I would only assess the patient using one or the other, not both. Please state your preference:

[r5] Maybe/I don’t know

Click here to see abbreviation definitions

Click here to see assumptions

Click here to see literature articles and abstracts

Click here to see survey 1 data

[Q11] In a patient who is in CR who was previously PET-CT+ at baseline, given the results of the CASIOPET Companion study, after HDT-ASCT, is the information you learn from MRD testing in bone marrow and PET/CT complementary?

Select one

[r1] Both provide important complementary data. I would use them together; the order of testing is not relevant

[r2] Both provide important complementary data, but I would test for MRD first and then do PET/CT

[r3] Both provide important complementary data, but I would do PET/CT first and then test for MRD

[r4] Both provide important data, but I would only assess the patient using one or the other, not both. Please state your preference:

[r5] Maybe/I don't know

Click here to see abbreviation definitions

Click here to see assumptions

Click here to see literature articles and abstracts

Click here to see survey 1 data

[Q12] In a patient who has undergone ASCT and who is currently receiving maintenance treatment, assuming you had access to multiple treatment options, would you recommend MRD testing in bone marrow if the patient:

[Q12a] Is in stringent CR

Select one

[r1] Yes, in most instances

[r2] Yes, under certain conditions; please specify:

[r3] No; it is rarely useful in such a patient

[r4] Stringent CR is not evaluated in my country

[r5] Maybe/I am not sure

[Q12b] Is in CR

Select one

[r1] Yes, in most instances

[r2] Yes, under certain conditions; please specify:

[r3] No; it is rarely useful in such a patient

[r5] Maybe/I am not sure

[Q12c] Has a VGPR (serum and urine paraprotein detectable by IF but not on electrophoresis) prior to HDT-ASCT

Select one

[r1] Yes, in most instances

[r2] Yes, under certain conditions; please specify:

[r3] No; it is rarely useful in such a patient

[r4] This IMWG definition of VGPR is not relevant in my country

[r5] Maybe/I am not sure

[Q12d] Has a VGPR (>90% reduction in serum paraprotein — although still detectable on electrophoresis — plus urine paraprotein level <100 mg/24h) prior to HDT-ASCT

Select one

Row:

[r1] Yes, in most instances

[r2] Yes, under certain conditions; please specify:

[r3] No; it is rarely useful in such a patient

[r5] Maybe/I am not sure

CR: IF- in serum and urine and PC in BM <5%; stringent CR: IF- in serum and urine and PC in BM <5% plus normal free light chain ratio and absence of clonal plasma cells, as assessed by IHC or immunofluorescence analysis *or by two-colour to four-colour flow cytometry*

Click here to see abbreviation definitions

Click here to see assumptions

Click here to see literature articles and abstracts

Click here to see survey 1 data

[Q13] In elderly patients (≥75 years old) undergoing maintenance treatment who are in CR and are transplant ineligible, given the results of the ALCYONE and MAIA trials, would you recommend testing MRD in bone marrow (noting that the patients are willing, and there are no logistical or technical barriers to testing)?

[r1] Yes; I would typically recommend testing for MRD

[r2] Yes, but only under certain circumstances; for instance, the patient is having mild toxicity to treatment and wants to cease therapy. If other circumstances are relevant, please specify:

[r3] No; current data on MRD are not sufficient to inform or influence my clinical decision-making at this point. For me to be more confident in using MRD results to inform or influence my practice, I would like to see strong clinical data with regard to the following:

[r4] No; other reason:

CR: IF- in serum and urine and PC in BM <5%

Click here to see abbreviation definitions

Click here to see assumptions

Click here to see literature articles and abstracts

Click here to see survey 1 data

[Q14] In patients (≤70 years old) undergoing maintenance treatment who are in CR and have chosen not to have HDT-ASCT or are transplant-ineligible, given the results of the ALCYONE, MAIA and IFM 2009 trials, would you recommend testing MRD in bone marrow? Question 10 of 29

Row:

[r1] Yes; I would typically recommend testing for MRD

[r2] Yes, but only under certain circumstances; for instance, the patient is having mild toxicity to treatment and wants to cease therapy. If other circumstances are relevant, please specify:

[r3] No; current data on MRD are not sufficient to inform or influence my clinical decision-making at this point. For me to be more confident in using MRD results to inform or influence my practice, I would like to see strong clinical data with regard to the following:

[r4] No; other reason:

CR: IF- in serum and urine and PC in BM <5%

Click here to see abbreviation definitions

Click here to see assumptions

Click here to see literature articles and abstracts

Click here to see survey 1 data

[Q15] In a newly diagnosed transplant-eligible patient who is currently undergoing maintenance and has achieved a VGPR defined as serum and urine paraprotein detectable by IF but not on electrophoresis, per MRD analysis in the IFM 2009 study trial, would you recommend testing for MRD in bone marrow if:

[Q15A] The patient completed induction but deferred/did not wish to undergo HDT-ASCT, and was directly started on the current maintenance therapy.

Select one

Row:

[r1] Yes, in most instances

[r2] Yes, under certain conditions; please specify:

[r3] No; it is rarely useful in such a patient

[r4] This IMWG definition of VGPR is not relevant in my country

[r5] Maybe/I am not sure

[Q15b] The patient completed induction, HDT-ASCT and was then started on the current maintenance therapy.

Select one

Row:

[r1] Yes, in most instances

[r2] Yes, under certain conditions; please specify:

[r3] No; it is rarely useful in such a patient

[r4] This IMWG definition of VGPR is not relevant in my country

[r5] Maybe/I am not sure

Click here to see abbreviation definitions

Click here to see assumptions

Click here to see literature articles and abstracts

Click here to see survey 1 data

[Q16] In a newly diagnosed transplant-ineligible patient who has completed induction, was started directly on maintenance and has achieved a VGPR, defined as serum and urine paraprotein detectable by IF but not on electrophoresis, would you recommend testing for MRD in bone marrow?

[r1] Yes, in most instances

[r2] Yes, under certain conditions; please specify:

[r3] No; it is rarely useful in such a patient

[r4] This IMWG definition of VGPR is not relevant in my country

[r5] Maybe/I am not sure

Click here to see abbreviation definitions

Click here to see assumptions

Click here to see literature articles and abstracts

Click here to see survey 1 data

[Q17] In a newly diagnosed transplant-eligible patient who is currently undergoing maintenance and has achieved a VGPR, defined as >90% reduction in serum paraprotein, although still detectable on electrophoresis, plus urine paraprotein level <100 mg/24 h, per MRD analysis in the IFM 2009 study trial, would you recommend testing for MRD in bone marrow if:

[Q17a] The patient completed induction but deferred/did not wish to undergo HDT-ASCT, and was started directly on the maintenance therapy

Select one

[r1] Yes, in most instances

[r2] Yes, under certain conditions; please specify:

[r3] No; it is rarely useful in such a patient

[r4] Maybe/I am not sure

[Q17b] The patient has completed induction, HDT-ASCT and was then started on the current maintenance therapy

Select one

[r1] Yes, in most instances

[r2] Yes, under certain conditions; please specify:

[r3] No; it is rarely useful in such a patient

[r4] Maybe/I am not sure

Click here to see abbreviation definitions

Click here to see assumptions

Click here to see literature articles and abstracts

Click here to see survey 1 data

[Q18] In a newly diagnosed transplant-ineligible patient, who has completed induction and has been started directly on maintenance and has achieved a VGPR, defined as >90% reduction in serum paraprotein, although still detectable on electrophoresis, plus urine paraprotein level <100 mg/24 h, would you recommend testing for MRD in bone marrow?

[r1] Yes, in most instances

[r2] Yes, under certain conditions; please specify:

[r3] No; it is rarely useful in such a patient

[r4] Maybe/I am not sure

Click here to see abbreviation definitions

Click here to see assumptions

Click here to see literature articles and abstracts

Click here to see survey 1 data

[Q19] Assuming the patient was willing and there are no logistical or practical

barriers, is MRD testing in bone marrow useful in elderly (≥75 years old) patients who are:

[Q19a] Frail and not in CR

[r1] Yes, in most instances

[r2] Yes, under certain conditions; please specify:

[r3] No; it is rarely useful in such patients

[r4] Maybe/I am not sure

[Q19b] Fit and not in CR

[r1] Yes, in most instances

[r2] Yes, under certain conditions; please specify:

[r3] No; it is rarely useful in such patients

[r4] Maybe/I am not sure

[Q19c] Frail and in CR

[r1] Yes, in most instances

[r2] Yes, under certain conditions; please specify:

[r3] No; it is rarely useful in such patients

[r4] Maybe/I am not sure

[Q19d] Fit and in CR

[r1] Yes, in most instances

[r2] Yes, under certain conditions; please specify:

[r3] No; it is rarely useful in such patients

[r4] Maybe/I am not sure

CR: IF- and PC in BM <5%; not in CR: serum/urine protein electrophoresis negativity for paraprotein, BM <5% but IF+

Click here to see abbreviation definitions

Click here to see assumptions

Click here to see literature articles and abstracts

Click here to see survey 1 data

[Q20] Is MRD testing in bone marrow useful in younger (<65 years old) patients who are:

[Q20a] Frail and not in CR

[r1] Yes, in most instances

[r2] Yes, under certain conditions; please specify:

[r3] No; it is rarely useful in such patients

[r4] Maybe/I am not sure

[Q20b] Fit and not in CR

[r1] Yes, in most instances

[r2] Yes, under certain conditions; please specify:

[r3] No; it is rarely useful in such patients

[r4] Maybe/I am not sure

[q20c]

[Q20c] Frail and in CR

[r1] Yes, in most instances

[r2] Yes, under certain conditions; please specify:

[r3] No; it is rarely useful in such patients

[r4] Maybe/I am not sure

[Q20d] Fit and in CR

[r1] Yes, in most instances

[r2] Yes, under certain conditions; please specify:

[r3] No; it is rarely useful in such patients

[r4] Maybe/I am not sure

CR: IF- and PC in BM <5%; not in CR: serum/urine protein electrophoresis negativity for paraprotein, BM <5% but IF+

Click here to see abbreviation definitions

Click here to see assumptions

Click here to see literature articles and abstracts

Click here to see survey 1 data

[Q21] Would you recommend MRD testing in bone marrow in a patient in the following relapse scenarios:

[Q21a] A patient who had received first-line therapy, responded and then had a non-refractory relapse, who has received second-line induction therapy and has now achieved a CR and is awaiting their deferred HDT-ASCT

[r1] Yes, in most instances

[r2] Yes, under certain conditions; please specify:

[r3] No; it is rarely useful in such patients

[r4] Maybe/I am not sure

[Q21b] A patient who had received first-line therapy, responded and then had a non-refractory relapse, who has received second-line induction therapy plus HDT-ASCT and is now receiving maintenance and has achieved a CR

[r1] Yes, in most instances

[r2] Yes, under certain conditions; please specify:

[r3] No; it is rarely useful in such patients

[r4] Maybe/I am not sure

[Q21c] A patient who had received first-line therapy, responded and then progressed while on therapy (refractory relapse), who has received second-line induction therapy and has achieved a CR, and is awaiting their deferred HDT-ASCT

[r1] Yes, in most instances

[r2] Yes, under certain conditions; please specify:

[r3] No; it is rarely useful in such patients

[r4] Maybe/I am not sure

[Q21d] A patient who had received first-line therapy, responded and then progressed while on therapy (refractory relapse), who has received second-line induction therapy plus HDT-ASCT and is now receiving maintenance and has achieved a CR

[r1] Yes, in most instances

[r2] Yes, under certain conditions; please specify:

[r3] No; it is rarely useful in such patients

[r4] Maybe/I am not sure

CR: IF- in serum and urine and PC in BM <5%

Click here to see abbreviation definitions

Click here to see assumptions

Click here to see literature articles and abstracts

Click here to see survey 1 data

Frequency of MRD testing

In this section, we ask that you answer questions under the additional assumption that you would test for MRD in the presented scenarios. This is so we can better understand your overall recommendations regarding MRD testing frequencies.

[Q22] A patient has undergone ASCT, how long would you typically recommend waiting post-ASCT before taking a fresh bone marrow sample for MRD assessment?

[r1] ≤3 months

[r2] 4–6 months

[r3] >6 months

[r4] I am not sure

Click here to see abbreviation definitions

Click here to see assumptions

Click here to see literature articles and abstracts

Click here to see survey 1 data

[Q23] A patient has undergone ASCT, is in CR, MRD-, and has recently (within the past year) been initiated on maintenance therapy. What is the minimum frequency at which you would recommend testing for sustained MRD status in bone

marrow?

[r1] ≤6 monthly

[r2] >6 monthly to annually

[r3] >Annually

[r4] I am not sure

CR: IF- in serum and urine and PC in BM <5%

Click here to see abbreviation definitions

Click here to see assumptions

Click here to see literature articles and abstracts

Click here to see survey 1 data

[Q24] A patient is in CR, is MRD+, has undergone ASCT and has recently (within the past year) been initiated on maintenance treatment. What is the minimum frequency at which you would recommend testing for sustained MRD status in bone marrow?

[r1] ≤6 monthly

[r2] >6 monthly to annually

[r3] >Annually

[r4] I am not sure

CR: IF- in serum and urine and PC in BM <5%

Click here to see abbreviation definitions

Click here to see assumptions

Click here to see literature articles and abstracts

Click here to see survey 1 data

[Q25] A patient is in CR, has undergone ASCT, was initiated on maintenance treatment two years ago and has been continuously MRD- for the duration of maintenance. What is the minimum frequency at which you would initially recommend testing for MRD in bone marrow?

[r1] ≤6 monthly

[r2] >6 monthly to annually

[r3] >Annually

[r4] I am not sure

CR: IF- in serum and urine and PC in BM <5%

Click here to see abbreviation definitions

Click here to see assumptions

Click here to see literature articles and abstracts

Click here to see survey 1 data

[Q26] A patient is in CR, has been MRD- for at least two years, and their treatment has recently (within the past year) been discontinued. What is the minimum frequency at which you would you initially recommend testing MRD in bone marrow?

[r1] ≤6 monthly

[r2] >6 monthly to annually

[r3] >Annually

[r4] I am not sure

CR: IF- in serum and urine and PC in BM <5%

Click here to see abbreviation definitions

Click here to see assumptions

Click here to see literature articles and abstracts

Click here to see survey 1 data

Continuous treatment as the norm

[Q27] Would you recommend testing for MRD in bone marrow periodically in a patient receiving continuous treatment who is in CR, to check if the patient has attained MRD negativity or to monitor depth of response over time? Question 23 of 29

[r1] Yes, in most instances

[r2] Yes, under certain conditions; please specify:

[r3] No; it is rarely useful in such a patient

[r4] Maybe/I am not sure

Click here to see abbreviation definitions

Click here to see assumptions

Click here to see literature articles and abstracts

Click here to see survey 1 data

[Q28] You answered "Yes" in the previous question; please indicate the minimum frequency at which you would initially recommend that MRD should be tested in this scenario?

Condition: **(Q27.r1 or Q27.r2)**

Row:

[r1] ≤6 monthly

[r2] >6 monthly to annually

[r3] >Annually

[r4] I am not sure

Click here to see abbreviation definitions

Click here to see assumptions

Click here to see literature articles and abstracts

Click here to see survey 1 data

Treatment discontinuation

[Q29] A patient is receiving continuous treatment, has been in CR for at least two years and has expressed desire to discontinue treatment due to adverse drug reactions. The patient has been MRD- in bone marrow and PET/CT- for the past two years. Would you support the patient's decision to cease therapy?

[r1] Yes; I would support the patient's decision to cease therapy

[r2] No; I would not support therapy discontinuation in the above scenario. If you selected this response, briefly describe the scenario(s), if any, in which you would support discontinuation

[r3] Maybe/I am not sure

CR: IF- in serum and urine and PC in BM <5%

Click here to see abbreviation definitions

Click here to see assumptions

Click here to see literature articles and abstracts

Click here to see survey 1 data

[Q30] What is the minimum length of time a patient in CR would need to be continuously MRD- (in bone marrow and on PET/CT) for you to support their decision to discontinue treatment?

[r1] ≤1 year as demonstrated on at least two occasions. Please indicate your recommended minimum interval between observations

[r2] >1–2 years as demonstrated on at least two occasions. Please indicate your recommended minimum interval between observations

[r3] >2 years as demonstrated on at least two occasions. Please indicate your recommended minimum interval between observations

[r4] I would not support a decision to discontinue treatment regardless. Please provide

your reasoning

CR: IF- in serum and urine and PC in BM <5%

Click here to see abbreviation definitions

Click here to see assumptions

Click here to see literature articles and abstracts

Click here to see survey 1 data

[Q31] In a patient who has ceased therapy and is willing/motivated to have bone marrow tests, how important is long-term MRD testing?

Drag the slider to a point on the scale.

Choice:

[ch1] 1 - Very important

[ch2] 2- Somewhat important

[ch3] 3 - Neither important nor unimportant

[ch4] 4 - Somewhat unimportant

[ch5] 5 - Not important

Click here to see abbreviation definitions

Click here to see assumptions

Click here to see literature articles and abstracts

Click here to see survey 1 data

Treatment re-initiation following discontinuation

[Q32] If relapse was detected in a previously MRD- standard-risk patient in whom treatment was discontinued, under ideal circumstances, please indicate the initial stage of relapse that you would prefer to re-initiate treatment (options are listed in alphabetical order).

[r1] Biochemical relapse

[r2] Clinical relapse

[r3] MRD+ relapse

Click here to see abbreviation definitions

Click here to see assumptions

Click here to see literature articles and abstracts

Click here to see survey 1 data

[Q33] If relapse was detected in a previously MRD- high-risk patient in whom treatment was discontinued, under ideal circumstances, please indicate the initial stage of relapse that you would prefer to re-initiate treatment (options are listed in alphabetical order)

[r1] Biochemical relapse

[r2] Clinical relapse

[r3] MRD+ relapse

Click here to see abbreviation definitions

Click here to see assumptions

Click here to see literature articles and abstracts

Click here to see survey 1 data

Factors that determine interest in testing for MRD

[Q34] In Survey 1, 85–90% of respondents stated that specific details concerning various patient or disease characteristics would either impact their motivation to test for MRD or they would be inclined to test MRD regardless. For us to better understand your overall recommendations, please rank the following patient or disease characteristics in order of their impact on influencing your decision to test for MRD. First = highest impact; last = least impact; options are currently listed in alphabetical order

Click or drag each item into a rank position.

Row:

[r1] Number of prior lines of therapy

[r2] Patient age

[r3] Patient ECOG performance status

[r4] Patient fitness/frailty

[r5] Patient R-ISS risk category

[r6] Presence of high-risk disease features

[r7] Time from initial diagnosis

[r8] Transplant eligibility

[r9] Type of prior relapse – aggressive/clinical versus indolent/biochemical

Click here to see abbreviation definitions

Click here to see assumptions

Click here to see literature articles and abstracts

Click here to see survey 1 data

Many thanks for your participation in this survey. Your responses to Survey 1 and Survey 2 (current survey) will be collated and used to draw conclusions and to determine if a third survey is required.

Appendix 3: Survey 1 case studies and results

Abbreviations used in this document

ASCT, autologous stem cell transplant; BM, bone marrow; CR, complete response; ECOG, Eastern Cooperative Oncology Group; HDT, high-dose therapy; IF, immuno-fixation; IHC, immunohistochemistry; IMWG; International Myeloma Working Group; MRD, measurable residual disease; MM, multiple myeloma; NGS, next generation sequencing; PC, plasma cells; PCR, polymerase chain reaction; PET/CT, positron-emission tomography/computed tomography; PS, performance status; R-ISS, Revised International Staging System; VGPR, very good partial response.

Survey 1: Use of MRD testing in MM in five case studies

Methods

As part of Survey 1, experts provided responses to questions around MM management strategy in five hypothetical MM cases. Experts were asked to make three assumptions. First, there was unrestricted access to testing facilities. Second, there were no barriers limiting the use of MRD testing. Third, MRD was assessed in bone marrow samples using either flow cytometry or PCR/NGS-based methodologies with limits of detection of at least 10^{-5}, where $<10^{-5}$ indicated MRD-negative (MRD-) status and 10^{-4}–10^{-3} indicated MRD-positive status (MRD+).

Results

Case study 1

- Patient description: The patient is a 61-year-old female with PS 0 and R-ISS II. Following four cycles of bortezomib, lenalidomide, and dexamethasone combination therapy, the patient achieves a CR, defined as IF-negative and <5% plasma cells in the BM (not stringent CR). She is positive on PET/CT at some sites. She is transplant-eligible, and you would like her to undergo HDT-ASCT
- Summary of responses: There was consensus agreement (89%) that respondents would proceed to ASCT regardless of patient's MRD results. Hence, only 51% of respondents would test for MRD prior to HDT-ASCT. If the patient achieved CR from HDT-ASCT, 75–82% of respondents agreed that the patient should undergo PET/CT imaging and MRD testing. Majority of respondents (93%) agreed that a negative status on both PET/CT and MRD test was important in indicating that the patient was disease-free. The

respondents agreed that MRD testing should occur ≤3 months post-ASCT (85%) and every 3 months to 1 year during maintenance therapy (76%). If the patient discontinued therapy, then 80% of respondents would assess for MRD every 3 months to 1 year (**Table 1**)

Table 1. Case study 1 questions and respondents' answers

	**Respondents (N=61)**
Would you recommend testing for MRD prior to HDT-ASCT?	
○ Yes	51%[a]
○ No	43%[b]
○ Maybe/I don't know	7%
You test and find that the patient is MRD+. What would you recommend?	
○ Treat with additional cycles of bortezomib, lenalidomide, and dexamethasone combination therapy until she is MRD-, before undergoing ASCT	11%
○ Continue directly to ASCT; MRD results would not impact my decision on moving directly to ASCT	**89%**
Your patient undergoes HDT-ASCT. You confirm that she is in 'conventional' CR (defined as IF-negative and <5% plasma cells in the BM), and you would like to initiate maintenance therapy	
○ Would you recommend re-assessing your patient's PET/CT status?	
• Yes	**75%**
• No	15%
• Maybe/I don't know	10%
○ Would you recommend confirming your patient's MRD status?	
• Yes	**82%**
• No	18%
• Maybe/I don't know	-
You decide to confirm MRD status. How long would you recommend waiting post-ASCT before taking a fresh BM sample for MRD assessment?	
○ <3 months	11%[c]
○ 3 months	74%[c]
○ 4 months	2%
○ 5 months	-
○ 6 months	5%

○ 12 months	2%
○ Other	7%
Is the information you learn from PET/CT and MRD complementary?	
○ Both provide important complementary data; would use together	**85%**
○ Both provide important data but I only need one or the other, not both	-
○ Would do PET/CT first and then, only if negative, would I test MRD	8%
○ Would test MRD first and then, only if negative, would I conduct PET/CT	7%
Your patient is initiated on maintenance therapy with lenalidomide post-ASCT	
○ Your patient remains in CR. You decide to assess MRD periodically. How frequently would you recommend assessing MRD during maintenance?	
• Every 3 months	5%[d]
• Every 6 months	33%[d]
• Every year	38%[d]
• I would not assess MRD regularly	16%
• Other[e]	8%
Your patient has been on maintenance therapy for two years. She remains in conventional CR defined as IF-negative and <5% plasma cells in BM (details of additional test results are given in the questions below). She is interested in discontinuing therapy as she has been experiencing some mild gastrointestinal-related side effects and is not keen to start alternative treatments. She wants to know if you would support her proposal to discontinue therapy.	
○ You would be content to support therapy if:	
• Scenario 1: Patient has been in CR for the past two years (no other tests are available)	16%
• Scenario 2: Patient is in CR with PET/CT- results for the past two years (patient has not been tested for MRD)	20%
• Scenario 3: Patient is in CR with MRD- results for the past two years (patient has not been tested for PET/CT status)	25%
• Scenario 4: Patient is in CR with MRD- and PET/CT- results for the past two years	74%
• Scenario 5: I would not support therapy discontinuation in any of the above scenarios.	23%[f]

If you would use MRD status in guiding whether you would support therapy discontinuation, what is the minimum amount of time that she would need to be continuously MRD- before you would support therapy discontinuation?	
○ One test indicating MRD- status (at a single time point) is sufficient	2%
○ A minimum of two tests demonstrating three months of MRD- is sufficient	7%[g]
○ A minimum of two tests demonstrating six months of MRD- is sufficient	16%[g]
○ A minimum of two tests demonstrating one year of MRD- is sufficient	21%[g]
○ A minimum of two tests demonstrating two years of MRD- is sufficient	31%[g]
○ Other	15%[h]
○ I would never use MRD results when making the decision to discontinue therapy	7%
○ I would never support therapy discontinuation in any circumstance	2%
The patient discontinues therapy	
○ At what frequency would you recommend that your patient be tested for MRD?	
• Every 3 months	7%[i]
• Every 6 months	34%[i]
• Every year	39%[i]
• I would not assess MRD regularly	8%
• I would not assess MRD in this patient	7%
• Other	5%
Provided she remains MRD-, for how long would you recommend continuing testing her for MRD?	
○ For a finite period of time	15%[j]
○ For as long as she is off treatment	56%
○ I would not assess MRD regularly	30%
Rank (1 = most important, 4 = least important) the following test results by their relative importance in indicating that your patient remains disease-free	
○ PET/CT- and MRD-	Average rank: 1.1
• 1	**93%**
• 2	3%
• 3	-
• 4	3%

○ MRD-	Average rank: 2.3
• 1	-
• 2	**80%**
• 3	15%
• 4	5%
○ PET/CT-	Average rank: 3.1
• 1	-
• 2	11%
• 3	66%
• 4	23%
○ IF-	Average rank: 3.5
• 1	7%
• 2	5%
• 3	20%
• 4	69%
At the 2-year assessment, the patient is MRD+	
○ At what point would you recommend to re-initiate therapy?	
• Immediately	7%
• Depends on MRD level	8%[k]
• Continue to monitor patient for sustained 'conventional' CR and not re-initiate therapy until patient has a clinical relapse	54%
• Other	31%[l]
You determine that your patient's risk status has progressed to high-risk. Would you use MRD to assist you in making therapeutic decisions in this patient?	
○ Yes	62%
○ No	25%
○ Maybe/I don't know	13%

Percentages in bold denote consensus was reached.

[a]Key reasons given by the respondents to recommend MRD testing: 1) May have prognostic significance; 2) To assess the efficacy of induction treatment; 3) There may be discrepancies between PET/CT and MRD.

[b]Key reasons given by the respondents not to recommend MRD testing: 1) Will proceed with transplant regardless of MRD result; 2) Patient is still positive on PET/CT so needs further treatment; 3) MRD-testing is more useful post-transplant.

[c]Clustered consensus to assess MRD within three months: 85% (sum of 11% and 74%).

[d]Clustered consensus to assess MRD every 3–12 months: 76% (sum of 5%, 33%, and 38%).

[e]Includes regular MRD testing only in patients who are high risk or cannot or refuse to receive maintenance therapy, or during clinical trials as needed.

[f]Some respondents noted that there are no data that would support treatment discontinuation based on MRD results. Others noted that if the patient was very motivated to stop treatment, then they would only support the patient in Scenario 4.
[g]Clustered consensus agreement that MRD negativity for three months to two years is needed to make decision: 75% (sum of 7%, 16%, 21%, and 31%).
[h]Respondents noted continuous MRD-negative status applies: 1) Only in patient-specific circumstances, such as patient request or severe side effects; 2) For as long as possible.
[i]Clustered consensus to assess MRD every 3–12 months: 80% (sum of 7%, 34%, and 39%).
[j]Responders' suggested times: 1 year (n=1), 2 year (n=1), 3 year (n=2), 5 years (n=5).
[k]Responders noted that a MRD threshold of 10^{-4} would prompt them to re-initiate therapy.
[l]Other details provided by the respondents are: 1) Monitor until significant biochemical/biological relapse (i.e., significant increase in M protein or FLC level); 2) Treatment depends on cytogenetic risk status, which can be deferred if not high risk.

Case study 2

- Patient description: Patient is a 75-year-old male who is newly diagnosed with PS 1. Following eight cycles of bortezomib, lenalidomide and dexamethasone induction therapy, the patient achieved ‘conventional’ CR (defined as IF- and <5% PC in BM). The patient is transplant-ineligible and currently on a lenalidomide maintenance therapy, which he is tolerating well
- Summary of responses: The respondents did not reach a consensus agreement on whether to test the patient for MRD; however, 62% of them would test for MRD. If the patient had undergone MRD testing and was MRD+ despite being in CR, 80% of respondents agreed that the patients should remain on maintenance therapy. There was no agreement on whether to continue maintenance therapy if the patient was MRD- (**Table 2**)

Table 2. Case study 2 questions and respondents’ answers

	**Respondents (N=61)**
Would you recommend testing your patient for MRD?	
○ Yes	62%[a]
○ No	28%[b]
○ Maybe/I don’t know	10%
Would you consider ceasing treatment? Rank (1 = strongly agree, 5 = strongly disagree)	
○ No; the patient is tolerating therapy	Average rating: 1.7
• 1	56%[c]
• 2	31%[c]
• 3	7%
• 4	5%
• 5	2%
○ I would use MRD as part of the consideration for ceasing treatment	Average rating: 2.9
• 1	18%
• 2	16%
• 3	31%
• 4	23%
• 5	11%
○ Yes; the patient is in CR and this is sufficient	Average rating: 4.1

• 1	2%
• 2	10%
• 3	7%
• 4	41%
• 5	41%
You test for MRD, and your patient is MRD+ despite being in CR. What would you recommend?	
○ Ceasing treatment; CR is sufficient	2%
○ Continuing the current maintenance regimen	**80%**
○ Modifying the current regimen or switching therapies to get the patient to MRD- status	10%
○ Other	8%[d]
You test for MRD, and your patient is MRD-. What would you recommend?	
○ Ceasing treatment	5%
○ Continue the current maintenance regimen	52%
○ Performing a re-evaluation after X months	39%[e]
○ Other	3%[f]

Percentages in bold denote consensus was reached.

[a]Key reasons for MRD testing are: 1) Relevant prognostic marker; 2) Helps assess depth of response; 3) Helps inform decision on potential to cease treatment.

[b]Key reasons for not recommending MRD testing: 1) MRD status will not change therapy recommendation; 2) Less relevant due to age of patient.

[c]Clustered consensus agreement to not cease treatment: 87% (sum of 56% and 31%).

[d]Recommendation would depend on individual patient and their quality of life on maintenance.

[e]Re-evaluation after 6 (n=12), 12 (n=11), and 24 (n=1) months.

[f]Recommendation would depend on level of risk and side effects.

Case study 3

- Patient description: Patient is a 65-year-old male with PS 2 and is transplant-eligible. The patient is receiving maintenance therapy with lenalidomide and has achieved a VGPR as determined by paraprotein level
- Summary of responses: The experts did not reach a consensus on whether to test the patient for MRD; however, 61% of them indicated they would not test for MRD. If the respondents decided to test for MRD, there was no agreement on the frequency of testing (**Table 3**)

Table 3. Case study 3 questions and respondents' answers

	Respondents (N=61)
Would you recommend testing your patient for MRD?	
○ Yes	25%[a]
○ No	61%[b]
○ Maybe/I don't know	15%
You decide to test your patient's MRD status. How frequently would you recommend that your patient be evaluated for MRD?	
○ Every 3 months	5%
○ Every 6 months	28%
○ Every 12 months	26%
○ There is no specific time point or frequency at which I use MRD; it depends on patient response	31%
○ Other	10%[c]
You test for MRD, and your patient is MRD-, despite only achieving a VGPR. What would you recommend?	
○ Retesting your patient's MRD status at the next scheduled MRD-testing appointment	38%
○ Retesting the patient's paraprotein level to see if it has reduced or is negative	38%
○ Other	23%[d]
○ I don't know	2%

[a]Key reasons for recommending MRD testing: 1) Patient could be MRD- despite VGPR; 2) there may be delayed clearance of the paraprotein; 3) MRD has prognostic value.
[b]Key reasons for not recommending MRD testing: 1) Testing is pointless based on description; 2) I would prefer to test in CR; 3) Paraprotein still present so would not consider stopping treatment; 4) MRD is likely to be positive.
[c]Respondents noted: 1) No perceived need; 2) Duration differs according to risk level.

[d]Respondents noted: 1) Test both MRD and paraprotein; 2) PET/CT or whole body MRI; 3) No need to re-test.

Case study 4

- Patient description: Patient is a 65-year-old male with PS 1, who has previously undergone ASCT. The patient recently relapsed and has since been started on and is currently receiving continuous treatment with daratumumab, bortezomib, and dexamethasone to ‘conventional’ CR (defined as IF- and <5% PC in BM). He has had one line of therapy previously and was not refractory
- Summary of responses: While not a consensus agreement, most respondents (69%) would test the patient for MRD. If the patient was MRD-, the respondents agreed (79%) to keep the patient on the current maintenance regimen (**Table 4**)

Table 4. Case study 4 questions and respondents’ answers

	Respondents (N=61)
Would you recommend testing your patient for MRD?	
○ Yes	69%[a]
○ No	20%[b]
○ Maybe/I don’t know	11%
Would you consider ceasing treatment? Rank (1 = strongly agree, 5 = strongly disagree)	
○ No; the patient is tolerating therapy	Average rating: 1.7
• 1	57%[c]
• 2	23%[c]
• 3	11%
• 4	7%
• 5	2%
○ I would use MRD as part of the consideration for ceasing treatment	Average rating: 2.5
• 1	28%
• 2	25%
• 3	21%
• 4	18%
• 5	8%
○ Yes; the patient is in CR, he is experiencing side effects and wants to stop therapy	Average rating: 2.7
• 1	11%
• 2	33%

• 3	36%
• 4	16%
• 5	3%
○ Yes; the patient is in CR and this is sufficient	Average rating: 4.4
• 1	2%
• 2	3%
• 3	5%
• 4	34%[d]
• 5	56%[d]
You test for MRD, and your patient is MRD+ despite being in CR. What would you recommend?	
○ Ceasing treatment; CR is sufficient	-
○ Continuing the current maintenance regimen	66%
○ Performing a re-evaluation after X months	30%[e]
○ Other	5%[f]
You test for MRD, and your patient is MRD-. What would you recommend?	
○ Ceasing treatment	10%
○ Continue the current maintenance regimen	**79%**
○ Other	11%[g]

Percentage in bold denote consensus was reached.

[a]Key reasons for recommending MRD testing: 1) Relevant prognostic marker; 2) Helps show depth of response; 3) Helps with decision on whether to cease treatment.

[b]Key reasons for not recommending MRD testing: 1) Will not lead to a change in treatment, regardless of result; 2) MRD utility is not validated at relapse.

[c]Clustered consensus agreement to not cease treatment: 80% (sum of 57% and 23%).

[d]Clustered consensus agreement to cease treatment: 90% (sum of 34% and 56%).

[e]Respondents noted re-evaluation after 3 (n=6), 6 (n=8), and up to 12 (n=3) months.

[f]Respondents noted they would consider changing therapy.

[g]Respondents noted that they would recommend: 1) Continue treatment until MRD- has been confirmed on subsequent tests, then consider ceasing treatment; 2) Confirming MRD status 6 months before ceasing treatment; 3) Re-testing after 12 months; 4) Based on patient's individual situation.

Case study 5

- Patient description: Your patient has previously undergone ASCT and is now undergoing maintenance therapy with lenalidomide, and is currently in CR; they want to cease maintenance therapy. Please indicate whether the following patient characteristics would impact your interest in knowing your patient's MRD status prior to the patient ceasing treatment
- Summary of responses: Consensus agreement was not reached on whether the respondents would want to know the patient's MRD status based on the different patient characteristics (e.g., patient/disease category, ECOG performance status, R-ISS risk) prior to supporting treatment discontinuation. However, the respondents who were interested in the patient's MRD status agreed that the interest was based on the assumption that the patient was ≤69 years old (88–91%), newly diagnosed (90%), transplant eligible (84%), in the high risk/high risk with R-ISS III category (86–91%), or on first line therapy (97%), or had experienced a relapse (88%; **Table 5**)

Table 5. Case study 5 questions and respondents' answers

	Respondents (N=61)
Would patient/disease category impact your interest in knowing your patient's MRD status prior to supporting their request to cease maintenance therapy?[a]	
○ Yes; I would want to know MRD status if the patient was within any of the following patient/disease category brackets	51%[b]
• Newly diagnosed	**90%**[c]
• Relapsed	55%[c]
• Transplant eligible	**84%**[c]
• Transplant ineligible	48%[c]
○ No; patient/disease category would not impact my interest in MRD status - I want to know MRD status regardless	39%[b]
○ No; patient/disease category would not impact interest in MRD status - MRD status would not impact treatment recommendations	10%
Would the patient's ECOG performance status or patient fitness/frailty impact your interest in knowing your patient's MRD status prior to supporting their request to cease maintenance therapy?	

○ Yes; I would want to know their MRD status if the patient was characterised as certain ECOG statuses or whether they were fit or frail[a]	64%[d]
• PS 0	74%[e]
• PS 1	72%[e]
• PS 2	49%[e]
• PS 3	21%[e]
• Fit	69%[e]
• Elderly/frail	38%[e]
○ No; ECOG status and patient fitness/frailty would not impact my interest in knowing their MRD status; I would want to know their MRD status regardless	21%[d]
○ No; ECOG status and patient fitness/frailty would not impact my interest in knowing their MRD status; MRD status would not impact my treatment recommendations	15%
Would patient age impact your interest in knowing their MRD status prior to supporting the patient in their request to cease maintenance therapy?	
○ Yes; I would want to know their MRD status if the patient was within certain age brackets[a]	54%[f]
• <40 years	**88%**[g]
• 40–49 years	**88%**[g]
• 50–48 years	**88%**[g]
• 60–69 years	**91%**[g]
• 70–79 years	73%[g]
• 80–89 years	12%[g]
• ≥90 years	9%[g]
○ No; patient age would not impact my interest in knowing their MRD status because I would want to know their MRD status regardless	31%[f]
○ No; patient age would not impact my interest in knowing their MRD status because MRD status would not impact my treatment recommendations	15%
Would patient R-ISS risk category impact your interest in knowing their MRD status prior to supporting the patient in their request to cease maintenance therapy?[a]	

○ Yes; I would want to know their MRD status if the patient was within any of the following risk categories	36%[h]
• High with R-ISS III	**91%**[i]
• High	**86%**[i]
• Standard	50%
○ No; risk category would not impact my interest in MRD status – I want to know MRD status regardless	51%[h]
○ No; risk category would not impact interest in MRD status – MRD status would not impact treatment recommendations	13%
Would patient treatment line/relapse history impact your interest in knowing their MRD status prior to supporting the patient in their request to cease maintenance therapy?[a]	
○ Yes; I would want to know their MRD status if the patient had the following treatment lines/relapse history	54%[j]
• First line	**97%**[g]
• Following first relapse	**88%**[g]
• Following second relapse	33%[g]
• Following third+ relapse	3%[g]
○ No; treatment line/relapse history would not impact my interest in MRD status – I want to know MRD status regardless	33%[j]
○ No; treatment line/relapse history would not impact interest in MRD status – MRD status would not impact treatment recommendations	13%

Percentages in bold denote consensus was reached.
[a]Respondents could select more than one option.
[b]Clustered consensus agreement that experts would want to know MRD status regardless of patient/disease category: 90% (sum of 51% and 39%).
[c]Based on n=31 respondents.
[d]Clustered consensus agreement that experts would want to know MRD status regardless of ECOG status or patient fitness/frailty: 85% (sum of 64% and 21%).
[e]Based on n=39 respondents.
[f]Clustered consensus agreement that experts would want to know MRD status regardless of age: 85% (sum of 54% and 31%).
[g]Based on n=33 respondents.
[h]Clustered consensus agreement that experts would want to know MRD status regardless of risk category: 87% (sum of 36% and 51%).
[i]Based on n=22 respondents.
[j]Clustered consensus agreement that experts would want to know MRD status regardless of treatment line/relapse history: 87% (sum of 54% and 33%).

Appendix 4: Survey 2 scenarios and results

Abbreviations used in this document

ASCT, autologous stem cell transplant; BM, bone marrow; CR, complete response; ECOG, Eastern Cooperative Oncology Group; HDT, high-dose therapy; HSCT, hematopoietic stem cell transplantation; IF, immuno-fixation; IHC, immunohistochemistry; IMWG; International Myeloma Working Group; MGUS, monoclonal gammopathy of undetermined significance; MRD, measurable residual disease; MM, multiple myeloma; NGS, next generation sequencing; OS, overall survival; PC, plasma cells; PCR, polymerase chain reaction; PET/CT, positron-emission tomography/computed tomography; PFS, progression-free survival; PS, performance status; R-ISS, Revised International Staging System; SPEP, serum protein electrophoresis; VGPR, very good partial response.

Survey 2: Use of MRD testing in different clinical scenarios

Methods

In responding to Survey 2, experts were asked to make four assumptions. First, there was unrestricted access to testing facilities. Second, there were no barriers limiting the use of MRD testing. Third, MRD was assessed in bone marrow samples using either flow cytometry or PCR/NGS-based methodologies with limits of detection of at least 10^{-5}; as such, MRD-positive indicates detection of at least one tumour cell in equal or less than 100,000 healthy cells. Fourth, patients who were MRD-positive had MRD values of no greater than once tumour cell in 1000 healthy cells.

Results

Questions were presented in the context of 16 clinical scenarios. Questions in the first five scenarios (**Scenarios 1–5**) arose from a lack of consensus agreement to the question regarding testing for MRD prior to HDT-ASCT in the 61-year-old female patient who was in CR and was transplant-eligible (**Appendix 3, Case Study 1**).

- **Scenario 1** described a patient who was in CR and was being considered for HDT-ASCT
    - Summary of responses: The respondents reached a consensus agreement (89%) that MRD testing should be recommended for the patient; however, there was no agreement on whether the testing should occur before or after HDT-ASCT

	Respondents (N=53)	Reasons
**Scenario 1:** A patient is in CR and is being considered for HDT-ASCT.		
• When would you recommend testing MRD in bone marrow?		
○ Only after induction therapy but before HDT-ASCT	-	
○ After induction therapy, before HDT-ASCT, and if the patient is MRD+ then	9%[a]	

also recommend testing after HDT-ASCT		Key reasons for recommending MRD testing both before and after HDT-ASCT:[c]<br>• Understand the disease burden / response<br>• Understand effects at different timepoints/ benchmarks
○ After induction therapy, before HDT-ASCT; also after HDT-ASCT, regardless of the MRD test result after induction therapy	21%[a]	
○ Only after HDT-ASCT, before starting maintenance	4%[a]	Key reasons for recommending MRD testing after HDT-ASCT:[d]<br>• ASCT guides treatment decision - MRD won't impact clinical decisions<br>• No data to support that ASCT could be avoided<br>• Look at MRD results (negative) under maintenance treatment / further monitoring<br>• Proceed to transplant irrespective of result
○ After HDT-ASCT, before starting maintenance treatment, and if the patient is MRD+ then also during maintenance	-	
○ After HDT-ASCT, before starting maintenance treatment; also, during maintenance regardless of the MRD test result after HDT-ASCT (before starting maintenance)	40%[a]	
○ Only after HDT-ASCT, during maintenance	-	
○ At multiple time points (not listed above) in this scenario; please state your recommended time points	15%[a,b]	
○ I would not recommend MRD in this situation	9%	Reasons for not recommending MRD testing in bone marrow:[e]<br>• No clinical consequence – status will not affect treatment decision
○ I am not sure	2%	

[a]Clustered consensus to recommend MRD testing: 89% (sum of 9%, 21%, 4%, 40%, and 15%)

[b]Other time points to test for MRD provided by the respondents: 1) Pre-ASCT, post-ASCT, during maintenance; 2) After induction, after HDT-ASCT and during the maintenance; 3) Before and after ASCT, at the end of consolidation and during maintenance, as long as the patient is in CR; 4) After induction, after ASCT, during maintenance; 5) At the moment of achieving CR, at the end of induction, before transplant (only in case of significant delay of transplant), after transplant, before consolidation/maintenance (in case of significant delay after transplant); 6) Before ASCT and 1 year later; 7) After induction before ASCT, 3 months after ASCT and every 6 months during maintenance; 8) After ASCT before starting maintenance, and every 6 months thereafter.

[c]Experts' reasons for recommending MRD testing both before and after HDT-ASCT: 1) The patient is in CR, if MRD negative maybe discussing delay ASCT. Controversial based on FORTE trial results, but might be preferred by some

patients, especially during COVID-19 times. MRD sustainability is important; 2) MRD before HSCT: I would study the level of response after the first step of therapy (induction). MRD after HSCT: I would study the effect of the consolidation with HSCT on the deepness of response. This could help to decide if another consolidation step could be necessary. These are the same steps used in clinical trials; 3) I want to know the effect of induction therapy and also evaluate MRD at different time points; 4) Ability to confirm MRD result. Pre-ASCT MRD result could sway patients from not considering transplant immediately. Look for sustained MRD negativity; 5) If the patient has MRD positivity before transplant, I'd like to know whether the response has improved, i.e., has become negative after ASCT. If the patient entered transplant already MRD-negative, I'd like to confirm that there have been no changes after ASCT and to be sure that the patient is still MRD negative before starting maintenance; 6) In order to assess patient response, since it is a prognosis marker; 7) MRD should be measured along with the treatment plan to evaluate response dynamics. Trials have shown that MRD can be improved after ASCT; 8) MRD negativity before ASCT could be prognostic; 9) Prior to HDT-ASCT: To assess the efficacy of induction therapy. After HDT-ASCT: To evaluate a possible need for consolidation therapy; MRD as benchmark before starting maintenance; 10) As soon as a patient is in CR we measure MRD to fine-tune the depth of response; 11) To have the best possible view on the disease burden.

[d]Experts' reasons for recommending MRD testing after HDT-ASCT: 1) I would proceed to ASCT regardless of the MRD status given this improves PFS and certainly will induce deeper MRD remissions; 2) To look at the possibility of MRD negativity under maintenance treatment; 3) No data supporting that we should not proceed to ASCT according to MRD; 4) No data so far to adapt intensive therapy based on pre-HDT MRD; 5) I would not tailor treatment before ASCT on the basis of MRD result; 6) Prognostic value. If MRD-negative during maintenance (at least 2 negative test in 6-12 months), it could be reasonable cease of therapy; 7) MRD-positive result pre-HSCT in a patient who is in CR is unlikely to change management currently as HSCT remains standard of care; 8) Actually, I would recommend testing for MRD after induction, then, if MRD-positive, also after HDT-ASCT and, finally, during maintenance (in the proposed multiple choice you did not have this hypothesis). Testing before and after ASCT is necessary in order to evaluate the role of melphalan 200 mg/m^2 in improving the quality of response. Testing after ASCT works as the baseline to monitor maintenance efficacy; 9) I still think that ASCT is the best option for eligible patients (data from the IFM, the EMN and the FORTE Trial), therefore MRD status would not impact on my decision of performing ASCT. I would recommend test after ASCT as this could guide treatment decision (further consolidation vs maintenance); 10) No evidence that ASCT could be avoided in post-induction MRD-negative patients; 11) I would also test MRD before ASCT, after ASCT, and during maintenance, independent of negativity/positivity of MRD, but that option was not there. My view is that sustained MRD negativity could in the future guide treatment decision (and maybe allowing discontinuation of maintenance); 12) Likely to have more MRD negativity after HDT as a baseline for future monitoring as best response; 13) Because I expect the highest level of MRD negativity to be after high-dose melphalan/ASCT. Regardless of MRD before high-dose melphalan I would continue with transplantation anyway; 14) I would recommend testing for MRD after HDT-ASCT, before starting maintenance treatment; I would also recommend testing for MRD during maintenance regardless of the MRD test result after HDT-ASCT (before starting maintenance). The rationale for this is ideally you would want >1 MRD-negative result to confirm true MRD negativity. Testing prior to ASCT is likely to have little value as you are likely to proceed to ASCT regardless; 15) It is prognostic at that time point based on trial data. At other time points is not clear and I don't see the point of wasting precious resource and lab time. Feel strongly it should not be used to direct treatment or at any other time point outside of clinical trials; 16) Testing the MRD before ASCT will not change my therapeutic attitude. From the FORTE study, we know that - even in MRD-negative patients, if there is no consolidation with ASCT patients will lose their MRD negativity; 17) Upfront treatment for transplant-eligible patients consists of induction therapy and intensification with high dose therapy-ASCT +/- consolidation therapy. If I administrate consolidation therapy after ASCT, I will evaluate MRD only after consolidation, before maintenance therapy, and at 6 and 12 months and then annually, considering patient is MRD-negative. According to MM XI, deepening the response > partial response before ASCT prolongs PFS, but there are no recommendation to deepen it if the patient achieved partial response and most probably MRD will be positive.

[e]Experts' reasons for not recommending MRD testing: 1) Not recommended by guidelines; 2) Still high-dose melphalan and ASCT are the recommended treatment so MRD status will not change treatment; 3) The patient will be having ASCT anyway as this is standard practice and therefore I would not check MRD as they are already in CR until day 100 post-transplant when it would be useful for ongoing monitoring; 3) Not useful for clinical decision, in my opinion. I will continue with HSCT; 4) It does not have any clinical consequence.

- **Scenarios 2–5** described a patient who was positive on PET/CT imaging and in CR. The patient was being considered for HDT-ASCT
    - Summary of responses:
        - The respondents agreed (77%) that PET/CT imaging should be conducted, but they did not agree on whether the imaging should occur before or after HDT-ASCT (**Scenario 2**)
        - Considering the results from the CASSIOPET Companion study[1], the respondents agreed (87–93%) that the status of MRD in the bone marrow and imaging on PET/CT are important before or after the patient had undergone HDT-ASCT (**Scenarios 3–5**)

	**Respondents (N=53)**
**Scenario 2:** A patient is in CR and is being considered for HDT-ASCT; the patient was PET/CT+ in some locations at baseline.	
• When would you recommend that the patient be tested using PET/CT?	
○ PET/CT before HDT-ASCT but not after	-
○ PET/CT after HDT-ASCT but not before	34%[a]
○ PET/CT both before and after HDT-ASCT	43%[a]
○ Test MRD in bone marrow before HDT-ASCT. If positive, consider clinical need and timing of PET/CT	11%
○ I would not perform PET/CT at all	9%
○ I am not sure	2%
**Scenario 3:** A patient in CR is being considered for HDT-ASCT; the patient was PET/CT+ in some sites at baseline.	
• Given results of the CASSIOPET Companion study[1], prior to HDT-ASCT, which test is more of a clinical priority in your opinion – MRD testing in bone marrow or PET/CT?	
○ MRD in bone marrow	32%
○ PET/CT	4%
○ Both are of equal priority; one does not outrank the other	60%
○ I am not sure	4%
**Scenario 4:** Indicate your level of agreement with the following statements. Consider the results of the CASSIOPET Companion study[1] in your answer.	
• In a patient who is in CR who was previously PET/CT+ at baseline, testing both MRD in bone marrow and PET/CT status prior to HDT-ASCT can provide clinically important information	Mean rating = 2.3
○ 1 (strongly agree)	30%

○ 2	38%
○ 3	11%
○ 4	15%
○ 5 (strongly disagree)	6%
• In a patient who is in CR who was previously PET/CT+ at baseline, testing both MRD in bone marrow and PET/CT status after HDT-ASCT can provide clinically important information.	Mean rating = 1.6
○ 1 (strongly agree)	60%[b]
○ 2	28%[b]
○ 3	2%
○ 4	6%
○ 5 (strongly disagree)	4%
**Scenario 5:** In a patient who is in CR who was previously PET/CT+ at baseline, given the results of the CASSIOPET Companion study[1]…	
• Prior to HDT-ASCT, what is your opinion on the use of MRD testing in bone marrow and PET/CT?	
○ I would use them together; the order of testing is not relevant	55%[c]
○ I would test for MRD first and then consider PET/CT	28%[c]
○ I would do PET/CT first and then consider testing MRD	4%[c]
○ I would only assess the patient using one or the other, not both. Please state your preference:	4%[d]
○ Maybe/I don't know	9%
• After HDT-ASCT, is the information you learn from MRD testing and PET/CT complementary?	
• Yes; I would use them together; the order of testing is not relevant	70%[e]
• Yes; I would test for MRD first and then consider PET/CT	21%[e]
• Yes; I would do PET/CT first and then consider testing MRD	2%[e]
• No; I would only assess the patient using one or the other, not both. Please state your preference:	2%[d]
• Maybe/I don't know	6%

[a]Clustered consensus to recommend PET/CT: 77% (sum of 34% and 43%).

[b]Clustered consensus agreement that both MRD testing and PET/CT provide important information: 88% (sum of 60% and 28%).

[c]Clustered consensus agreement to perform both MRD testing and PET/CT imaging: 87% (sum of 55%, 28%, and 4%).

[d]Respondents indicated that they would prefer MRD testing.
[e]Clustered consensus agreement that information from MRD testing and PET/CT is complementary: 93% (sum of 70%, 21%, and 2%).

Questions in clinical **Scenarios 6–16** arose from a lack of consensus agreement on MRD testing in the Case Studies 2–4 (**Appendix 3**) that involved 65–75-year-old male patients who achieved either VGPR or CR with treatment.

- **Scenario 6** described a patient who had undergone ASCT and was receiving maintenance treatment
  - Summary of response: The respondents reached consensus agreement that they would recommend MRD testing if the patient was in stringent CR (89%) or CR (87%)

	Respondents (N=53)
**Scenario 6:** In a patient who has undergone ASCT and who is currently receiving maintenance treatment, assuming you had access to multiple treatment options, would you recommend MRD testing in bone marrow if the patient…	
• Is in stringent CR	
○ Yes, in most instances	**89%**[a]
○ Yes, under certain conditions; please specify	8%[a,b]
○ No, it is rarely useful in such a patient	-
○ Maybe/I am not sure	4%
○ This IMWG definition not relevant in my country	-
• Is in CR	
○ Yes, in most instances	**87%**[c]
○ Yes, under certain conditions; please specify	8%[b,c]
○ No, it is rarely useful in such a patient	4%
○ Maybe/I am not sure	2%
○ This IMWG definition not relevant in my country	-
• Has a VGPR (serum and urine paraprotein detectable by IF but not on electrophoresis) prior to HDT-ASCT	
○ Yes, in most instances	40%
○ Yes, under certain conditions; please specify	13%[d]
○ No, it is rarely useful in such a patient	43%
○ Maybe/I am not sure	2%
○ This IMWG definition not relevant in my country	2%
• Has a VGPR (>90% reduction in serum paraprotein – although still detectable on electrophoresis – plus urine paraprotein level (<100 mg/24h) prior to HDT-ASCT	
○ Yes, in most instances	28%
○ Yes, under certain conditions; please specify	11%[e]

○ No, it is rarely useful in such a patient	58%
○ Maybe/I am not sure	2%
○ This IMWG definition not relevant in my country	-

Percentages in bold denote consensus was reached

[a]Clustered consensus to assess MRD: 97% (sum of 89% and 8%).

[b]Other conditions to recommend MRD testing provided by the respondents: 1) In clinical trial only; 2) If patient has high-risk disease who experienced intense relapse.

[c]Clustered consensus to assess MRD: 95% (sum 87% and 8%).

[d]Other conditions to recommend MRD testing provided by the respondents: 1) After 1 year to understand the evolution of response; 2) In clinical trial only; 3) Not before D100 post-HSCT; 4) Previous MGUS, daratumumab treatment-IgG; 5) VGPR maybe misleading if on daratumumab; 5) If PET/CT was negative.

[e]Other conditions to recommend MRD testing provided by the respondents: 1) In clinical trial only; 2) If patient has achieved CR; 3) If PET/CT was negative.

- **Scenarios 7 and 8** described patients (≤70 or ≥75 years old) who were undergoing maintenance treatment, in CR, and either transplant-ineligible or unwilling to undergo HDT-ASCT
  - Summary of responses: 76% of respondents agreed that they would test for MRD in some or most circumstances if the patient was ≤70 years old and not ≥75 years old

	Respondents (N=53)
**Scenario 7:** In elderly patients (≥75 years old) undergoing maintenance treatment who are in CR and are transplant ineligible, given the results of the ALCYONE[2] and MAIA[3] trials, would you recommend testing MRD in bone marrow (noting that the patients are willing, and there are no logistical or technical barriers to testing)?	
○ Yes; I would typically recommend testing for MRD	51%
○ Yes, but only under certain circumstances; for instance, the patient is having mild toxicity to treatment and wants to cease therapy. If other circumstances are relevant, please specify	17%[a]
○ No; current data on MRD are not sufficient to inform or influence my clinical decision-making at this point. For me to be more confident in using MRD results to inform or influence my practice, I would like to see strong clinical data with regard to the following	28%[b]
○ No; other reason	4%[c]

[a]Respondents' conditions to recommend MRD testing or opinion regarding stopping treatment: 1) Depends on distance to the hospital; 2) I would stop treatment if MRD-negative; 3) Perhaps if considering dose modifications to the regimen; 4) If the patient is fit; 5) If a >75 years old patient receiving daratumumab maintenance progressed and has a clinical relapse and pomalidomide with bortezomib/cyclophosphamide is the only alternative therapy we have. I would not recommend carfilzomib; 6) In general do not feel there is data to stop treatment, but this may change in time.

[b]Key clinical data respondents would like to be more confident in using MRD: 1) Outcomes (including OS and PFS) of patients who are MRD-negative or have sustained MRD negativity (>6 or >12 months) who stopped the treatments; 2) PFS / OS improvement based on MRD adapted strategy; 3) Treatment decision making based on MRD status; 4) Early MRD relapse knowledge having an impact on long-term survival.

[c]Other reasons to not recommend MRD testing provided by the respondents: 1) No data to support stopping treatment if MRD-negative; 2) It does not modify my current approach.

	Respondents (N=53)
**Scenario 8:** In patients (≤70 years old) undergoing maintenance treatment who are in CR and have chosen not to have HDT-ASCT or are transplant-ineligible, given the results of the ALCYONE[2], MAIA[3] and IFM 2009[4] trials, would you recommend testing MRD in bone marrow?	
○ Yes; I would typically recommend testing for MRD	68%[a]
○ Yes, but only under certain circumstances; for instance, the patient is having mild toxicity to treatment and wants to cease therapy. If other circumstances are relevant, please specify:	8%[a,b]
○ No; current data on MRD are not sufficient to inform or influence my clinical decision-making at this point. For me to be more confident in using MRD results to inform or influence my practice, I would like to see strong clinical data with regard to the following:	19%[c]
○ No; other reason	6%[d]

[a]Clustered consensus to assess MRD: 76% (sum of 68% and 8%).

[b]Other conditions to test for MRD provided by the respondents: 1) If making decision to change or stop treatment; 2) If considering ASCT for the patient - would be more inclined to consider ASCT if patient is MRD-positive.

[c]Key clinical data respondents would like to be more confident in using MRD: 1) Data on stopping treatment in MRD-negative patients; 2) Data on treatment decision making based on MRD status; 3) Relevance of MRD status to OS or PFS; 4) Impact of MRD-positive relapse on long-term OS.

[d]Other reasons to not recommend MRD testing provided by the respondents: 1) No data on stopping according to MRD; 2) It does not modify my current approach; 3) Clinical trial only.

- **Scenario 9** described a newly diagnosed transplant-eligible patient who was undergoing maintenance and had achieved a VGPR defined as serum and urine paraprotein detectable by IF but not on electrophoresis
    - Summary of responses: While not a consensus agreement, over half of the respondents (61–70%) would recommend MRD testing under some or most conditions whether or not the patient had undergone HDT-ASCT during maintenance therapy

	**Respondents (N=53)**
**Scenario 9:** In a newly diagnosed transplant-eligible patient who is currently undergoing maintenance and has achieved a VGPR defined as serum and urine paraprotein detectable by IF but not on electrophoresis, per MRD analysis in the IFM 2009 study trial[4], would you recommend testing for MRD in bone marrow if…	
• The patient completed induction but deferred/did not wish to undergo HDT-ASCT, and was directly started on the current maintenance therapy	
○ Yes, in most instances	55%
○ Yes, under certain conditions; please specify	6%[a]
○ No; it is rarely useful in such a patient	36%
○ This IMWG definition of VGPR is not relevant in my country	4%
○ Maybe/I am not sure	-
• The patient completed induction, HDT-ASCT and was started on the current maintenance therapy	
○ Yes, in most instances	62%
○ Yes, under certain conditions; please specify	8%[b]
○ No; it is rarely useful in such a patient	25%
○ This IMWG definition of VGPR is not relevant in my country	4%
○ Maybe/I am not sure	2%

[a]Other conditions to test for MRD according to the respondents: 1) If the patient achieves CR during maintenance; 2) If PET/CT was carried out.

[b]Other conditions to test for MRD according to the respondents: 1) If the patient achieves CR during maintenance; 2) Clinical trial only; 3) If patient has high risk genetics; 4) If PET/CT was performed.

- **Scenario 10** described a newly diagnosed transplant-ineligible patient who had completed induction, was started directly on maintenance and had achieved a VGPR
    - Summary of responses: Opinion was split on whether to perform MRD testing in this patient (47% Yes: 49% No)

	Respondents (N=53)
**Scenarios 10:** In a newly diagnosed transplant-ineligible patient who has completed induction, was started directly on maintenance and has achieved a VGPR, defined as serum and urine paraprotein detectable by IF but not on electrophoresis, would you recommend testing for MRD in bone marrow?	
○ Yes, in most instances	34%
○ Yes, under certain conditions; please specify:	13%[a]
○ No; it is rarely useful in such a patient	49%
○ This IMWG definition of VGPR is not relevant in my country	2%
○ Maybe/I am not sure	2%

[a]Other conditions to test for MRD according to the respondents: 1) If mild toxicity is reported; 2) If the patient achieves CR during maintenance; 3) Only if MRD leads to intervention leading to better PFS; 4) Not in frail patients; 5) Only in clinical trial; 6) If PET/CT was carried out.

- **Scenario 11** described a newly diagnosed transplant-eligible patient who was undergoing maintenance and had achieved a VGPR
    - Summary of responses: While not a consensus agreement, over half of the respondents (55–62%) would recommend MRD testing under some or most conditions regardless of whether the patient had undergone HDT-ASCT during maintenance therapy

	Respondents (N=53)
**Scenarios 11:** In a newly diagnosed transplant-eligible patient who is currently undergoing maintenance and has achieved a VGPR, defined as >90% reduction in serum paraprotein, although still detectable on electrophoresis, plus urine paraprotein level <100 mg/24 h, per MRD analysis in the IFM 2009 study trial[4], would you recommend testing for MRD in bone marrow if…	
• The patient completed induction but deferred/did not wish to undergo HDT-ASCT, and was started directly on the maintenance therapy	
○ Yes, in most instances	40%
○ Yes, under certain conditions; please specify:	15%[a]
○ No; it is rarely useful in such a patient	45%
○ Maybe/I am not sure	-
• The patient has completed induction, HDT-ASCT and was then started on the current maintenance therapy	
○ Yes, in most instances	49%
○ Yes, under certain conditions; please specify:	13%[b]
○ No; it is rarely useful in such a patient	38%
○ Maybe/I am not sure	-

[a]Other conditions to test for MRD according to the respondents: 1) The patient shows chemo sensitivity; 2) BM <5% PC; 3) If criteria for testing are standardized; 4) Daratumumab IgG; 5) MRD could guide treatment decision if negative becomes positive; 6) If the patient achieves CR during maintenance; 7) Clinical trial only; 8) If PET/CT was carried out.

[b]Other conditions to test for MRD according to the respondents: 1) Criteria must be standardized; 2) Daratumumab IgG; 3) If the patient achieves CR during maintenance; 4) Clinical trial only; 5) If PET/CT was carried out.

- **Scenario 12** described a newly diagnosed transplant-ineligible patient, who completed induction and started directly on maintenance. The patient achieved a VGPR, defined as >90% reduction in serum paraprotein, although still detectable on electrophoresis, plus urine paraprotein level <100 mg/24 h
    - Summary of response: Over half of the respondents (55%) indicated they would not recommend MRD testing for this patient

	Respondents (N=53)
**Scenario 12:** In a newly diagnosed transplant-ineligible patient, who has completed induction and has been started directly on maintenance and has achieved a VGPR, defined as >90% reduction in serum paraprotein, although still detectable on electrophoresis, plus urine paraprotein level <100 mg/24 h, would you recommend testing for MRD in bone marrow?	
○ Yes, in most instances	26%
○ Yes, under certain conditions; please specify:	17%[a]
○ No; it is rarely useful in such a patient	55%
○ Maybe/I am not sure	2%

[a]Other conditions to test for MRD according to the respondents: 1) If bone marrow PC < 5%; 2) If young patient and expected long OS and Daratumumab IgG treatment; 3) If the patient achieves CR during maintenance; 4) If using MRD to inform treatment decision to achieve/maintain MRD-negative status; 5) If the patient is not frail; 6) During maintenance; 7) Clinical trial only; 8) If patient experiences toxicity and wishes to stop maintenance; 9) If the patient is <75 years of age.

- **Scenario 13** addressed whether MRD testing is useful in elderly (≥75 years old) patients
    - Summary of responses:
        - The respondents reached consensus agreement (89%) that MRD testing would not be useful if the patient was frail and not in CR
        - 81% of the respondents agreed that MRD testing is useful if the patient was fit and in CR

	Respondents (N=53)
**Scenario 13:** Assuming the patient was willing and there are no logistical or practical barriers, is MRD testing in bone marrow useful in elderly (≥75 years old) patients who are:	
• Frail and not in CR	
○ Yes, in most instances	9%
○ Yes, under certain conditions; please specify:	2%[a]
○ No; it is rarely useful in such patients	**89%**
○ Maybe/I am not sure	-
• Fit and not in CR	
○ Yes, in most instances	11%
○ Yes, under certain conditions; please specify:	19%[b]
○ No; it is rarely useful in such patients	70%
○ Maybe/I am not sure	-
• Frail and in CR	
○ Yes, in most instances	23%
○ Yes, under certain conditions; please specify:	25%[c]
○ No; it is rarely useful in such patients	53%
○ Maybe/I am not sure	-
• Fit and in CR	
○ Yes, in most instances	62%[d]
○ Yes, under certain conditions; please specify:	19%[d,e]
○ No; it is rarely useful in such patients	13%
○ Maybe/I am not sure	6%

Percentages in bold denote consensus was reached.

[a]Other condition in which MRD testing is useful according to the respondents: Clinical trial only.

[b]Other conditions in which MRD testing is useful according to the respondents: 1) If in VGPR; 2) If VGPR SPEP negative; 3) If VGPR or better and BM <5% PC; 4) If mild toxicity is reported; 5) Clinical trial only; 6) If patient has high risk disease; 7) Only if patient is not in CR due to IF-positive; 8) If patient is fit to consider subsequent treatment; 9) If treatment decision is dependent on result.

[c]Other conditions in which MRD testing is useful according to the respondents: 1) May use MRD to inform treatment discontinuation; 2) If treatment is interrupted due to toxicity; 3) If patient is frail but receiving active therapy; 4) If study

results on MRD based treatment de-escalation are available; 5) If there is question about maintaining treatment. 6) Clinical trial only

[d]Clustered consensus agreement that MRD testing is useful: 81% (sum of 62% and 19%).

[e]Other conditions in which MRD testing is useful according to the respondents: 1) If there is toxicity on maintenance; 2) If expected longer survival; 3) If deciding to stop treatment; 4) Optimizing/adapting treatment to keep the patient in CR; 5) If treatment is interrupted due to toxicity; 6) If study results on MRD based treatment de-escalation are available; 7) If patient is well enough to consider further combination therapies and has high risk disease; 8) Clinical trial only; 9) If patient has high risk disease; 10) If there is alternative therapy.

- **Scenario 14** addressed whether MRD testing is useful in younger (<65 years old) patients
  - Summary of responses:
    - The respondents reached consensus agreement (79%) that MRD testing would not be useful if the patient was frail and not in CR
    - The respondents reached consensus agreement (83%) that MRD testing would be useful in most instances if the patient was fit and in CR

	Respondents (N=53)
**Scenario 14:** Is MRD testing in bone marrow useful in younger (<65 years old) patients who are…	
• Frail and not in CR	
○ Yes, in most instances	11%
○ Yes, under certain conditions; please specify	8%[a]
○ No; it is rarely useful in such patients	**79%**
○ Maybe/I am not sure	2%
• Fit and not in CR	
○ Yes, in most instances	17%
○ Yes, under certain conditions; please specify	25%[b]
○ No; it is rarely useful in such patients	58%
○ Maybe/I am not sure	-
• Frail and in CR	
○ Yes, in most instances	36%
○ Yes, under certain conditions; please specify	34%[c]
○ No; it is rarely useful in such patients	26%
○ Maybe/I am not sure	4%
• Fit and in CR	
○ Yes, in most instances	**83%[d]**
○ Yes, under certain conditions; please specify	8%[d,e]
○ No; it is rarely useful in such patients	6%
○ Maybe/I am not sure	4%

Percentages in bold denote consensus was reached.

[a]Other conditions in which MRD testing is useful according to the respondents: 1) If patient is in VGPR; 2) If there is an impact on treatment decision; 3) Clinical trial only; 4) If fit enough to consider further treatment.

[b]Other conditions in which MRD testing is useful according to the respondents: 1) If patient is in VGPR or better; 2) VGPR SPEP negative; 3) Daratumumab IgG; 4) If there is doubt about CR criteria; 5) Clinical trial only; 6) If patient is not in CR due to IF-positive; 7) If decision on treatment based on MRD result.

[c]Other conditions in which MRD testing is useful according to the respondents: 1) If making decision about treatment intensification; 2) If patient will receive maintenance; 3) Toxicity/cease treatment; 4) Toxicity on maintenance; 5) If

frail because of MM and expected to recover; 6) Frail and receiving active therapy; 7) If study results on MRD based treatment de-escalation should are available; 8) If patient has high risk disease and is well enough to consider further combination therapies at MRD relapse; 9) If there is an impact on treatment decision; 10) Clinical trial only; 11) If considering treatment discontinuation; 12) If testing will have an impact on the treatment ; 13) If fit enough to consider further treatment.

[d]Clustered consensus agreement that MRD testing is useful: 91% (sum of 83% and 8%).

[e]Other conditions in which MRD testing is useful according to the respondents: 1) If patient is on maintenance, after ASCT; 2) If making treatment decision; 3) If study results on MRD based treatment de-escalation are available; 4) Clinical trial only.

- **Scenario 15** described a patient who had responded to first-line therapy, but then had a non-refractory relapse
    - Summary of responses: 82% of respondents would recommend MRD testing in some or most instances if the patient received second-line induction therapy plus HDT-ASCT and achieved CR while on maintenance therapy

	Respondents (N=53)
**Scenario 15:** A patient who had received first-line therapy, responded and then had a non-refractory relapse, who has…	
• Received second-line induction therapy and has now achieved a CR and is awaiting their deferred HDT-ASCT. Would you recommend MRD testing in bone marrow?	
○ Yes, in most instances	64%
○ Yes, under certain conditions; please specify	8%[a]
○ No; it is rarely useful in such patients	21%
○ Maybe/I am not sure	8%
• Received second-line induction therapy plus HDT-ASCT and is now receiving maintenance and has achieved a CR. Would you recommend MRD testing in bone marrow?	
○ Yes, in most instances	74%[b]
○ Yes, under certain conditions; please specify	8%[b,c]
○ No; it is rarely useful in such patients	15%
○ Maybe/I am not sure	4%

[a]Other conditions to test for MRD according to the respondents: 1) If the ASCT is delayed for some reason and there are maintenance options; 2) To maintain the CR prior to stem cell transplant; 3) Only if it can impact the decision to proceed to ASCT; 4) Clinical trial only.

[b]Clustered consensus to assess MRD: 82% (sum of 74% and 8%).

[c]Other conditions to test for MRD according to the respondents: 1) Whether patient is fit enough to consider further combination therapy and has high-risk disease; 2) Before starting maintenance; 3) Clinical Trial only; 4) If patient experiencing toxicity and wishes to stop maintenance.

- **Scenario 16** described a patient who responded to first-line therapy, but had refractory relapse while on therapy
    - Summary of responses: The respondents achieved consensus (75%) that they would recommend MRD testing if the patient received second-line induction therapy plus HDT-ASCT and achieved CR while on maintenance therapy

	Respondents (N=53)
**Scenario 16:** A patient who had received first-line therapy, responded and then progressed while on therapy (refractory relapse), who has…	
• Received second-line induction therapy and has achieved a CR, and is awaiting their deferred HDT-ASCT. Would you recommend MRD testing in bone marrow?	
○ Yes, in most instances	64%
○ Yes, under certain conditions; please specify	6%[a]
○ No; it is rarely useful in such patients	25%
○ Maybe/I am not sure	6%
• Received second-line induction therapy plus HDT-ASCT and is now receiving maintenance and has achieved a CR. Would you recommend MRD testing in bone marrow?	
○ Yes, in most instances	**75%**
○ Yes, under certain conditions; please specify	6%[b]
○ No; it is rarely useful in such patients	15%
○ Maybe/I am not sure	4%

Percentages in bold denote consensus was reached.

[a]Other conditions to test for MRD according to the respondents: 1) If the ASCT is delayed for some reason and there are maintenance options; 2) Only if it has an impact on the decision to proceed to ASCT; 3) Clinical trial only.

[b]Other conditions to test for MRD according to the respondents: 1) Before starting maintenance; 2) Clinical trial only; 3) Patient experiencing toxicity and wishes to stop maintenance.

References

1. Moreau P, Attal M, Hulin C, et al. Bortezomib, thalidomide, and dexamethasone with or without daratumumab before and after autologous stem-cell transplantation for newly diagnosed multiple myeloma (CASSIOPEIA): A randomised, open-label, phase 3 study. *Lancet* 2019; **394**(10192): 29-38.
2. Mateos MV, Dimopoulos MA, Cavo M, et al. Daratumumab plus bortezomib, melphalan, and prednisone for untreated myeloma. *N Engl J Med* 2018; **378**(6): 518-28.
3. Facon T, Kumar S, Plesner T, et al. Daratumumab plus lenalidomide and dexamethasone for untreated myeloma. *N Engl J Med* 2019; **380**(22): 2104-15.
4. Perrot A, Lauwers-Cances V, Corre J, et al. Minimal residual disease negativity using deep sequencing is a major prognostic factor in multiple myeloma. *Blood* 2018; **132**(23): 2456-64.
